# Risk and Resilience: How Is the Health of Older Adults and Immigrant People Living in Canada Impacted by Climate- and Air Pollution-Related Exposures?

**DOI:** 10.3390/ijerph182010575

**Published:** 2021-10-09

**Authors:** McKenzie H. Tilstra, Ishwar Tiwari, Leigh Niwa, Sandra Campbell, Charlene C. Nielsen, C. Allyson Jones, Alvaro Osornio Vargas, Okan Bulut, Bernadette Quemerais, Jordana Salma, Kyle Whitfield, Shelby S. Yamamoto

**Affiliations:** 1 School of Public Health, Edmonton Clinic Health Academy, University of Alberta, Edmonton, AB T6G 1C9, Canada; ishwar@ualberta.ca (I.T.); scampbel@ualberta.ca (S.C.); ccn@ualberta.ca (C.C.N.); shelby.yamamoto@ualberta.ca (S.S.Y.); 2 Faculty of Rehabilitation Medicine, University of Alberta, Edmonton, AB T6G 2G4, Canada; lniwa@ualberta.ca (L.N.); allyson.jones@ualberta.ca (C.A.J.); 3 Faculty of Medicine and Dentistry, University of Alberta, Edmonton, AB T6G 2R7, Canada; osornio@ualberta.ca (A.O.V.); bernadette.quemerais@ualberta.ca (B.Q.); 4 Faculty of Education, University of Alberta, Edmonton, AB T6G 2G5, Canada; bulut@ualberta.ca; 5 Faculty of Nursing, Edmonton Clinic Health Academy, University of Alberta, Edmonton, AB T6G 1C9, Canada; sjordana@ualberta.ca; 6 School of Urban and Regional Planning, Faculty of Science, University of Alberta, Edmonton , AB T6G 2E3, Canada; kw16@ualberta.ca

**Keywords:** climate change, air pollution, health-impacts, older adults, immigrants

## Abstract

Background: In the rapidly shifting Canadian climate, an ageing population, and increased migration, a greater understanding of how local climate and air pollution hazards impact older adults and immigrant populations will be necessary for mitigating and adapting to adverse health impacts. Objectives: To explore the reported health impacts of climate change and air pollution exposures in older adults and immigrant people living in Canada, identify known factors influencing risk and resilience in these populations and gaps in the literature. Methods: We searched for research focused on older adults and immigrants living in Canada, published from 2010 onward, where the primary exposures were related to climate or air pollution. We extracted data on setting, exposures, health outcomes, and other relevant contextual factors. Results and Discussion: We identified 52 eligible studies, most focused in Ontario and Quebec. Older people in Canada experience health risks due to climate and air pollution exposures. The extent of the risk depends on multiple factors. We found little information about the climate- and air pollution-related health impacts experienced by immigrant communities. Conclusions: Further research about climate- and air pollution-related exposures, health, and which factors promote or reduce resiliency in Canada’s older adults and immigrant communities is necessary.

## 1. Introduction

Climate change has been called “the defining issue” for public health in the 21st century [1], and air pollution the single most significant environmental health risk by the WHO [2]. Health impacts of these exposures are felt intensely at a local scale, depending on the socioenvironmental context. Canada is warming two-fold faster than the global average [3], which is expected to amplify adverse health impacts via multidimensional exposure pathways, including changing weather patterns leading to extreme temperatures, drought, floods, and wildfires [3,4,5]. Likewise, there is a well-established link between air pollution and adverse health outcomes. Health Canada has estimated that 15,300 premature deaths in Canada each year are attributable to air pollution [6].

Complex relationships between climatic variables (i.e., temperature, precipitation, humidity and wind) and air pollution can also impact human health. For example, the adverse health effects of heatwaves can be compounded by poor air quality [7]. Changes in climate associated with increasing frequency, season, and length of wildfires in Canada can also contribute to air pollution and adverse respiratory, cardiovascular, injury and mental health events [5].

Climate change is expected to exacerbate existing health risks and introduce new ones. However, the high degree of spatial variability in climatic and air pollution exposures across Canada (due to expansive geography, land use, and industry, combined with diverse population demographics and social environments) presents a multifaceted challenge for public health in Canada. Moreover, the health burden may fall disproportionately on specific populations due to intersections of physiological, social, and environmental factors [8,9,10].

Two populations that may face greater health vulnerability due to climate change and air pollution are older adults and immigrants [10]. In Canada, this has ramifications from a public health perspective given the older adult population continues to grow [11] and immigrants comprised over one-fifth of the country’s population in 2016 (a share that is projected to increase) [12,13].

Both younger and older adults will face health impacts from climate change and air pollution. However, some older adults may face physiological changes, multimorbidity, mobility limitations, lack of awareness, social isolation, poor housing conditions, and inadequate access to health and welfare services, which could limit their capacity to cope with these effects compared to younger persons [7,14,15,16,17,18].

While Canadian immigrants may arrive healthier than native-born Canadians, this effect diminishes with time [19]. Furthermore, it is not clear whether this effect exists for older immigrants at all [19]. The health of immigrant populations may also be impacted by socioeconomic differences, living arrangements, cultural and/or language barriers, and occupational exposures (e.g., outdoor) that can exacerbate climate change effects [19,20,21,22].

Although recent reviews have focused on climate change resilience and adaptation concerning health, research has not explicitly focused on these population’s exposures, sensitivities, and adaptive capacities [21,23]. It is important to explore the impact of climate change and air pollution on specific growing vulnerable groups such as older adults and immigrant communities to inform interventions to reduce disproportionate impacts.

## 2. Materials and Methods

A scoping review method was used to map the literature on climate- and air pollution-related health outcomes in Canada’s older adults and immigrant populations. The protocol for this review was developed a priori for transparency and replicability and was registered on the Open Science Framework [24]. The review framework was informed and guided by the methodology for scoping reviews defined by Arksey and O’Malley et al. [25]. We followed the research methodology and reporting standards for scoping reviews defined by the Preferred Reporting Items for Systematic Reviews and Meta-Analyses Extension for Scoping Reviews (PRISMA-ScR) [26].

### 2.1. Identifying the Research Question

We established the following questions to examine and synthesize the range, extent, and nature of published literature in Canada with respect to climate, air pollution, and health in older adult and immigrant populations:

1. What are the reported health impacts of climate- and air pollution-related exposures in older adults and immigrants in Canada?
2. Are there socioeconomic and contextual characteristics that impact the relationship between these exposures and adverse health outcomes in these populations?

### 2.2. Identifying Relevent Studies

A search was constructed and executed by a health research librarian (SC) on nine electronic databases: PROSPERO, OVID Medline, OVID EMBASE, OVID Global Health, OVID PsycInfo, Cochrane Library (CDSR and Central), EBSCO CINAHL, Proquest Dissertations and Theses Global and SCOPUS using a controlled vocabulary (e.g., MeSH, Emtree) and keywords representing the concepts “vulnerable populations” and “climate change” and “health impacts” and “Canada” (Table S1). Databases were searched from 2010 to June 2020. No other limits were applied. Detailed searches are available in the supplementary materials.

### 2.3. Study Selection

Citations (3684) were imported to the Covidence systematic review program and then duplicates (1141) were removed. A two-staged screening process was conducted by two independent reviewers (κ = 0.59) (MT, IT). Titles and abstracts were first screened using a stacked screening form. At the second stage, the full text of each article was then screened. To be included, articles had to: (1) focus on exposure to climatic or air pollution variables; (2) discuss health impacts; (3) report results related to older adults and/or immigrant populations; (4) focus on Canadian populations; and (5) have been published as a peer-reviewed journal article between the beginning of 2010 and June 2020 to capture the most recent available literature following the publication of a report on the health vulnerabilities due to climate change in Canada [10]. Citations that did not meet all of these criteria were excluded. We focused on climate- and air pollution-related exposures, including extreme temperatures, wildfires, icy conditions, and ambient air pollution. Health impacts were considered through the lens of the WHO definition of health as “a state of complete physical, mental, and social wellbeing and not merely the absence of disease or infirmity” [27]. Older adults were defined as anyone 65 years and older. We applied the Statistics Canada definition of an immigrant as referring “to a person who is, or ever has been, a landed immigrant or permanent resident” [28]. Throughout the screening process, reviewers met to resolve conflicts related to study inclusion and exclusion.

### 2.4. Charting the Data

Articles that met the inclusion criteria were included in the scoping review results. An extraction form capturing the following data was developed: year of publication, study time period, study location(s) and setting, study methodology, climatic and air pollution exposure variable(s) and assessment methods, health outcome(s), socioeconomic characteristic(s) studied, additional variables and interaction(s) explored, and whether sex/gender differences were considered in the study. Two independent reviewers (MT and LN) manually extracted half of the articles, each using this standardized form vetted by the research team. Three sample articles were jointly extracted to validate the form before continuing with the remaining articles independently.

### 2.5. Collating, Summarizing, and Reporting the Results

Descriptive statistics were conducted in Stata^®^ (Version 16) (StataCorp LLC., College Station, TX, USA) and Microsoft Excel^®^ (2016) (Microsoft Corp., Redmond, WA, USA) and data were narratively synthesized. The details of studies included in this review are presented in tables.

## 3. Results

### 3.1. Profile of Studies

**Figure 1:**
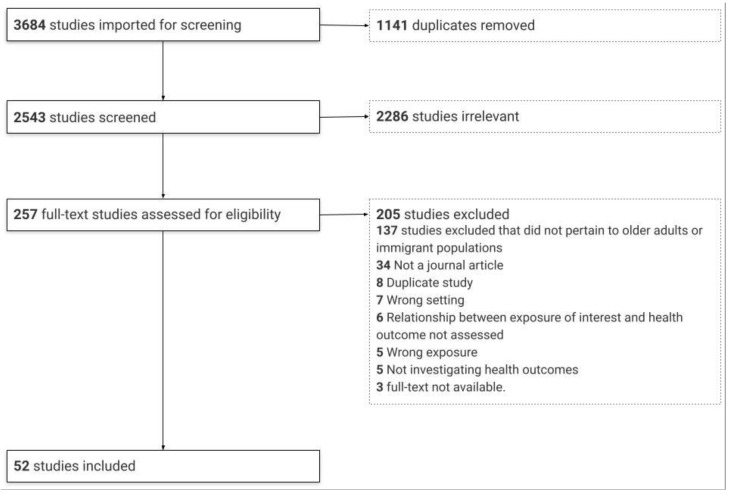
Flow diagram of the study selection process.

**Figure 2:**
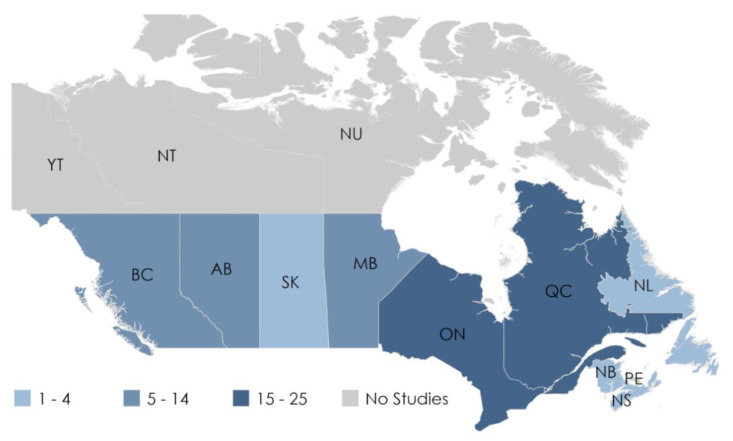
The frequency populations from each province were represented in the studies. A number of studies were specifically focused on populations in Ontario (20), Quebec (15), British Columbia (9), and Alberta (2), while the remaining studies included specific populations from multiple provinces.

A total of 2543 citations were identified for screening, of which 257 full texts were reviewed. Fifty-two papers were included (Figure 1). Table 1 summarizes the key characteristics of the studies included in this review. A detailed tabular summary is available in the supplementary material (Table S2). The articles included data ranging from 1980 and 2015, with the longest study period covering 30 years. Populations in Ontario (25 of 52; 48%) and Quebec (20 of 52; 38%) were studied most frequently. The majority of studies in Ontario were population based and covered all residents living in Ontario, differentiated by region and urban settings when possible. No studies investigating locations in Prince Edward Island, Yukon, Northwest Territories, or Nunavut, the least populous regions, reported results on either older adult or immigrant populations. Figure 2 summarizes the locations of the included studies.

All of the studies used quantitative research methods. The most common study designs were ecological (15 of 52; 29%), cohort (14 of 52; 27%), or variations of case–control studies (12 of 52; 23%). The remaining publications included cross-sectional studies (3 of 52; 6%), panel studies (2 of 52; 4%), non-randomized experimental studies (2 of 52; 4%), one randomized control trial, one case-only analysis, and one review.

Nearly all papers (51 of 52; 98%) examined age differences and reported results pertaining to older adults. Several articles included a measure of immigrant population density; however, only two papers presented health impacts in Canada’s immigrant populations.

Most of the articles included air pollution and meteorological parameters broadly grouped based on the primary exposure of interest. One article was included in both categories because it reported on the health impacts of each as the primary exposure. Across all articles, the health impacts studied were: cardiovascular; respiratory; mortality; morbidity (i.e., emergency department visits); diabetes; cancer; fall-related injuries; neurological; mental health; renal; musculoskeletal; and ocular-related health outcomes.

### 3.2. Meteorological Parameters

Twenty-two papers (22 of 52; 42%) examined health impacts due to meteorological parameters as primary exposures, including temperature, humidity, snow depth, rainfall, and flooding. The majority of these studies (12 of 22; 55%) were conducted in urban settings [29,31,32,33,35,36,40,41,42,44,45,50] or covered broader areas in Ontario [30,37,48,49], Quebec [38,43], and British Columbia [34] (7 of 22 (31%)), while two studies were conducted in lab settings [46,81]. In studies that involved wider geographic areas, authors generally controlled for the region of residence (i.e., urban or rural). One study was a review covering the health impacts of flooding in Canada [39].

#### 3.2.1. Health Impacts of Meteorological Parameters on Older Adults

Broad health impacts, such as non-accidental mortality or overall morbidity (i.e., total health-services use), made up nearly half of the publications, followed by cardiovascular outcomes, musculoskeletal, respiratory, mental, renal, ocular-related health impacts, and diabetes. Evidence suggests that older adults in Canada face health risks due to meteorological exposures, including weather temperature, precipitation-related variables, and flooding (Table 2). Still, results were not consistent in terms of whether they experience increased risk compared to other age groups.

##### Mortality

Eight studies (8 of 22; 36%) reported results pertaining to the effects of temperature and heatwaves on non-accidental or all-cause mortality [33,34,35,36,37,38,39,50]. Mortality was associated with temperature in older adults [35,38,50], and in some studies, they exhibited higher levels of risk than younger age groups [33,36]. Benmarhnia et al. found that temperature–mortality relationship differences by age were dependent on the contrast measure used, with a percentile method of comparison (i.e., 95th vs. 50th percentiles) proving more sensitive in detecting differences [33]. Within the older adult age stratum, it is not clear whether certain age subgroups of older adults experience greater risk to heat events, as sometimes “very old” (i.e., ≥75 years) subgroups demonstrated greater risk, while in other cases, “younger old” (i.e., 65–74 years) were at greater risk. One review found that while Canada tends to experience low flood-mortality rates overall, older adults may be at greater mortality risk [39].

##### Overall Morbidity

Five studies (5 of 22; 24%) presented results on the effect of temperature on overall morbidity [38,44], perceived state of health during heat events [45], and other physiological measures of health [46,81]. One prospective cohort study of older adults found that significant emergency department presentations and hospitalizations occurred more frequently when the daily maximum temperature was ≥ 30 °C [44]. Yet, another study found that the prevalence of health impacts, measured as perceived overall state of health during “very hot and humid days,” increased with advancing age only up until 65 years, after which it decreased [45]. Two non-randomized experimental studies found that older adults had more difficulty identifying ambient temperature decreases and did not perform as well as younger persons across a battery of tests, with heat posing a greater thermal challenge [46] and cold impairing dexterity and grip [47].

##### Cardiovascular Health Outcomes

Four studies (4 of 22; 18%) reported results on the effects of meteorological variables on cardiovascular-related health outcomes [29,30,37,50]. Extreme temperatures were associated with cardiovascular mortality [37,50], and heart failure [29,37,50]. Heat specifically was associated with cardiac arrest [37] and stroke in elderly individuals taking oral anticoagulants [30]. While Bai et al. found that acute myocardial infarction may be exclusively associated with cold temperatures, more moderate temperatures were responsible for a far greater burden of coronary heart disease and stroke hospitalizations [30]. Generally, there was little evidence for age effects.

##### Respiratory Health Outcomes

Three studies (3 of 22; 14%) reported results on the effects of meteorological exposures on respiratory health outcomes in older adults. In two studies, authors found that temperature was linked to respiratory mortality [37,50]; however, Krstic et al. suggest that there was no strong evidence that those ≥65 years of age were at greater risk than other age groups [50]. Respiratory impacts related to flood exposures were also identified as a possible health hazard for older adults [39].

##### Fall-Related Injuries

Three studies (3 of 22; 14%) described the effect of meteorological variables on fall-related injuries [40,41,42]. Decreasing temperatures, and increased snow depth, and the number of snowy days appeared to be associated with increased hip fracture rates across all age groups [41,42], hours of daylight, atmospheric air pressure, and rainfall depth were important for older females with similar results for males, except for rainfall depth [41,42]. Another study found statistically significant increases in fall-related injuries among older adults following freezing rain warnings, except for hip fractures [40].

##### Mental Health Outcomes

Four studies (4 of 22; 18%) reported results on mental health impacts in older adults. Higher temperatures were significantly associated with mental and behavioral disorders in older adults. Wang et al. found greater effects in the elderly [32]. In contrast, Bélanger et al. measured the perceived state of mental and physical health during heat events. They found that the prevalence of poor mental and physical health was lower in older adults compared to younger ages [45]. A review identified older adults as generally at greater risk from flooding, with mental illness as a potential health impact [39]. One study did not report any difference by age [31].

##### Renal Health Outcomes

Two studies (2 of 22; 9%) reported results on renal health impacts in older adults [48,49]. Increased heat exposure was associated with an increased risk of hospital admissions for acute kidney infection in older adults (IQR 74–85 years of age) [48] and emergency department visits for renal colic [49]. However, the latter association was stronger for those between the ages of 40 and 69 years.

##### Ocular Health Outcomes

The results of one study indicated that while acute exposure to elevated temperatures is associated with a higher likelihood of traction retinal detachment, the effect was stronger in those <75 years of age [43].

##### Diabetes

A single study investigated the effect of hot and cold temperatures on diabetes-related mortality, though the results were null [37].

#### 3.2.2. Health Impacts in Immigrant Populations

One cross-sectional survey study found that those born outside of Canada reported a lower prevalence of perceived physical and mental health impacts during very hot and humid days [45].

### 3.3. Air Pollution

Most publications (31 of 52; 59%) included in this review examined health impacts due to air pollution as a primary exposure. Most were conducted in urban settings across Canada (21 of 31; 68%) or covered wider areas within Ontario [58,59,72,73,74,79,80], Alberta [55], or British Columbia [66] (9 of 31; 29%). Researchers generally controlled for the region of residence (i.e., urban or rural) when covering broader geographies. One panel study specifically focused on a rural population in Ontario [62].

#### 3.3.1. Health Impacts of Air Pollution in Older Adults

The relationship between air pollution and cardiovascular and respiratory health outcomes was most frequently studied in older adults. However, cancer, neurological health outcomes, diabetes, mortality, and musculoskeletal health outcomes were also investigated (Table 3). Generally, air pollution had a deleterious impact on health but effects were somewhat specific to pollutants and outcomes.

##### Cardiovascular Health Outcomes

Sixteen studies (16 of 31; 52%) assessed the impact of air pollution on cardiovascular outcomes in older adults. The cardiovascular outcomes included total cardiovascular and circulatory mortality, atrial fibrillation, acute myocardial infarction, stroke [54,55,56,58,59,60], total cardiovascular morbidity [66], coronary heart disease [57], congestive heart failure [59], hypertension [65], and other subclinical cardiovascular measures [62,63,64]. Taken together, these sources indicate a relationship between air pollution and older adult cardiovascular health, but findings across older age groups were not always consistent and depended on the pollutant and condition of interest.

Total cardiovascular and circulatory mortality in older adults was associated with air pollutant exposure (PM_2.5_ [68,69], CO, SO_2_, NO_2_ [67,69], and O_3_ [61,68]), except for one cohort study [69]. Regarding the effect of age, evidence was mixed, with some research reporting higher levels of risk among older adults relative to younger age groups and others reporting little effect by age.

Atrial fibrillation and related acute cardiovascular events, such as acute myocardial infarction (AMI) and stroke incidence in older adults, were also linked to PM_2.5_ [56,58,59], NO_2_ [55,58] O_3_, and redox-weighted averages of NO_2_ and O_3_ (O_x_) exposure [58,59]. While not consistent across all studies, greater effect sizes in older adults were generally observed for PM_2.5_ exposure and AMI [59], especially in those with hypertension [56]. Findings were similar for O_3_ and O_x_ exposure and stroke [58]. Likewise, the Air Quality Health Index (AQHI) demonstrated a stronger association with acute ischemic stroke in older seniors [54].

Relationships were observed between air pollution and cardiovascular morbidity. For example, wildfire smoke exposure, assessed via PM_10_, was linked to total cardiovascular morbidity in those between the ages of 40 and 50 years and those ≥ 80 years [66]. Exposure to PM_2.5_, NO_2_, O_3_, and O_x_ was positively associated with congestive heart failure (CHF) incidence in older adults [59] and ultrafine particles (<0.1 µm in diameter) (UFP) and NO_2_ were associated with hypertension [65]. In both cases, effect sizes were smaller in older ages compared to younger ages. The AQHI was also associated with sub-clinical adverse cardiorespiratory effects in those over 55 years of age [62,63]. Another study also found associations between air pollution and daily cardiovascular measures and markers of oxidative stress in adults aged 55–81 [64]. No age differences were observed for associations between coronary heart disease (CHD) morbidity and black carbon exposure [57].

##### Respiratory Health Outcomes

Eleven studies (11 of 31; 35%) investigated the effects of air pollution on respiratory health outcomes in older populations. These publications examined general respiratory mortality [50,67,68,69] and morbidity [66,80], COPD mortality and morbidity [78], asthma [66,76,77,79], and pneumonia [75]. All but three of these studies [76,77,80] found that air pollution was associated with older adult respiratory health; however, results were not clear about whether older adults were at greater risk of adverse respiratory health outcomes than populations under the age of 65.

While O_3_, PM_2.5_, black carbon, and NO_2_ have been linked to respiratory mortality across populations in Canada, stratification by age generally revealed that when associations were observed, they tended to be weaker for older ages compared to populations under the age of 65 [50,67,68,69,78]. In one study, significant estimates between PM_2.5_ and respiratory mortality were observed in those ≥75 years of age [68]. Results were similar in terms of total respiratory morbidity [80], COPD [78], and acute bronchitis and upper respiratory infection diagnoses [66]. One case–control study including only those ≥65 years of age also found that long-term exposure to increased levels of NO_2_ and PM_2.5_ was independently associated with pneumonia hospitalization [75].

Likewise, PM_10_, PM_2.5_, SO_2_, O_3,_ and the Air Quality Health Index were associated with asthma morbidity across populations but not always observed in older age groups. The level of risk in older adults often depended on the specific age subgroup [66,76,77]. For example, associations with PM_10_ were observed for those between the ages of 60 and 70 years but not those between 70 and 80 [66], while another study found that those ≥60 years of age generally had greater risk ratios than adults <65 years [79].

##### Cancer

Four studies (4 of 31; 13%) assessed the effects of air pollution on cancer [51,52,53,69]. Articles generally assessed older populations and found that air pollutants, including UFP, NO_2_, SO_2_, and O_3_, increased the risk of certain cancers. Exposure to NO_2_ [53] and UFP [52,53] showed positive, albeit insignificant, associations with incident postmenopausal breast cancer in older women and strong positive associations were observed between NO_2_, O_3_, and SO_2_ and cancer mortality in older adults [69]. While significant associations were found between NO_2_ and incident prostate cancer in men, minimal age differences were observed [51].

##### Neurological Health Outcomes

Three studies (3 of 31; 10%) investigated the impacts of air pollution on Parkinson’s disease (PD) and dementia [72,73,74]. Air pollution appears to have a negative impact on the neurological health of older adults; however, results were varied as to which pollutants pose a risk. In terms of incident PD, studies found significant associations with PM_2.5_ [73,74] and O_3_ [74]; however, there were conflicting results with respect to NO_2_ [73,74]. Dementia was associated with exposure to PM_2.5_ and NO_2_ [72,73], but not O_3_ [72].

##### Diabetes

Two studies (2 of 31; 6%) investigated the effects of air pollution and diabetes [65,69]. Studies found that PM_2.5_, NO_2_, CO, and SO_2_ were associated with mortality in older adults [69] and UFP particles and NO_2_ with diabetes morbidity [65]. In terms of morbidity, effect sizes in older adults were smaller compared to younger age groups.

##### Mortality

Four studies (4 of 31; 13%) investigated the effects of air pollution on non-accidental mortality in several provinces [67,68,69,70]. Associations were observed between PM_2.5_ [68,69], NO_2_ [67,69,70], O_3_ [68,70], SO_2_, and CO [69,70] and non-accidental mortality; however, age effects were not consistent. While somewhat pollutant dependent, older adults generally exhibited an increased risk of mortality with air pollution exposure, but this risk was not necessarily greater than other age groups.

##### Musculoskeletal Outcomes

A single study assessed the effects of air pollution on rheumatoid arthritis (RA) [71]. While the authors observed that RA was associated with proximity to traffic, neither traffic-related air pollution (TRAP) nor noise was responsible for the associations, which were stronger for those <65 years of age.

#### 3.3.2. Health Impacts in Immigrant Populations

One Montreal-based study investigated the effects of air pollution on health in immigrant populations. Parent et al. found that exposure to NO_2,_ as a measure of TRAP, was associated with an increased risk of incident prostate cancer in the general population, but that risks were higher amongst men in the third quartile distribution of recent immigrants [51].

### 3.4. Factors Influencing the Climate– and Air Pollution–Health Relationships

Sociodemographic, socioeconomic, and environmental factors may also modify air pollution and meteorological impacts on older adult health. Some studies did explicitly explore the intersection of these factors in older adults (e.g., lower-income older adults). Sociodemographic variables included sex and the proportion of immigrants in a neighborhood. Socioeconomic indicators included measures of income, education, and employment. Environmental variables included seasonality, region of residence (i.e., urban vs. rural), and population density. Comorbidities were frequently an important factor concerning the health impacts of climate and air pollution exposures in older adults.

#### 3.4.1. Sociodemographic Characteristics

Sex and the proportion of immigrants per neighborhood were the variables most commonly adjusted for in analyses. Over half of the studies explored sex differences, but the results were varied. Sex differences were observed more frequently when assessing the effects of meteorological parameters, including heat and precipitation, on health. However, one sex was not consistently at higher risk than the other across studies. Generally, differences in risk by sex depended on specific exposures. The proportion of immigrants in neighborhoods was adjusted for in several air pollution studies, though rarely in meteorological studies.

Other sociodemographic characteristics, including visible minority status, ethnicity, and marital status, in addition to comorbidity and behavioral factors, appeared to influence health risks from environmental exposures. Laverdière et al. found that high social participation in older adults was strongly protective in relation to adverse heat-related events. At the same time, disability or requiring assistance for daily living activities was a risk factor [44].

#### 3.4.2. Socioeconomic Characteristics

Indicators of socioeconomic status (SES) were frequently considered in studies where air pollution was the primary exposure (16 of 31; 52%), compared to a smaller number of meteorologically-focused studies (7 of 22; 32%). The most common variables used to account for SES were income, education, and employment. While most studies adjusted for these factors, a number of those that stratified according to SES reported that lower-income, less-than high school diploma education and unemployment rates were associated with higher levels of risk for adverse health outcomes, especially with respect to air pollution. Low income was the main indicator of SES and a predictor of adverse health outcomes with meteorological exposures. Indicators were often at the neighborhood-level, rather than the individual level. Some studies noted little effect or confounding by SES in terms of air pollution, and in one study, high SES was associated with increased cardiovascular hospitalization [57].

#### 3.4.3. Environmental Characteristics

A number of studies adjusted for seasonality as an important covariate in terms of the effect of both air pollution and meteorological factors; however, interactions between seasonality and these exposures were studied less frequently. Similarly, authors found that the risk of mortality in older adults due to air pollution appeared to be modified by different large-scale weather systems (synoptic weather type) [70]. Place of residence at both regional and local scales also influenced the effect of meteorological and air pollution exposures on health. For example, stronger associations between climatic and air pollution exposures and adverse health impacts were observed for those living in urban areas compared to rural [73,74]. Some research also observed within-city variation [67,68]. Evidence also suggests that population density may be relevant to health risk in older adults [34,36]. Henderson et al. found that the effect of temperature was markedly higher for those aged <75 years in three of four ecologically distinct geographical areas in British Colombia, except for the most densely populated coastal region [34].

## 4. Discussion

In the rapidly shifting Canadian climate [3] with an ageing population [11] and continued migration [12], we found that older adult health is at risk due to extreme weather, increasing temperatures, and air pollution. However, we identified a gap in our knowledge of how these exposures impact immigrant health. Furthermore, not all areas across Canada have been investigated to the same extent with people residing the Prairies, Maritimes, and Territories being studied less than the more populous Ontario and Quebec, if at all.

### 4.1. Evidence for Older Adult Health Risk due to Climate- and Air Pollution-Related Exposures in Canada

Several studies investigated climate- and air pollution-related health risks in older people living in Canada. There is compelling evidence that older adult health is adversely affected by these exposures. Specifically, older adults were at risk of cardiovascular, respiratory, mortality and overall morbidity outcomes due to meteorological and air pollution exposures. Relationships with musculoskeletal, mental health, renal, and ocular health impacts were only observed for meteorological exposures, and cancer, neurological disorders, and diabetes impacts were only observed for air pollution in older adults. It is worth noting that the health burden of these exposures may be underestimated since the frequent use of mortality and forms of acute care as measures of health tend to capture more severe health impacts.

Significant associations among older adults were typically observed for specific exposures and conditions (e.g., O_3_ and stroke), which differed by age group. In terms of the impact of PM_10_ on respiratory health, for instance, significant associations were only detected among those between 60 and 70 years of age but not among older age strata [79], and similar results were observed for some meteorological parameters. These findings support that climate and air pollution stressors overall pose a risk to older adults’ mental and physical health in Canada and are consistent with several other works focused on higher-income countries [18,82,83,84,85].

The integrative review by Leyva et al. identified that older adults do not necessarily perceive themselves to be suffering a greater risk of health impacts. However, they bear a disproportionate burden of health impacts due to climate stressors [18]. Likewise, we found that older adults tended to face greater health risks from heat effects. Experimental studies identified they had more difficulty identifying temperature changes and performed poorer tests in both heat and cold conditions than younger people [46,47]. However, those ≥65 years of age experienced a decreased prevalence of *perceived* health impacts during “very hot and humid days” compared to those under 65 [45].

However, overall the differences in health effects between older and younger persons in Canada are unclear. Over one-third of studies that looked at different age groups reported stronger associations in older adults than in younger adults [32,33,39,42,46,47,54,55,56,68,70,79] while others did not [31,37,38,43,61,71,78]. Many articles reported mixed results depending on specific outcomes and contextual factors. While many of the effects of climate change and air pollution are not restricted to older adults, factors like limited mobility and pre-existing medical conditions can render them more severe [85]. Future work focused on this question could help to establish how older adults differ from younger adults within this context.

All of the studies included in this review employed quantitative methods and were predominantly epidemiological. While epidemiological studies are necessary for quantifying the risk posed, qualitative methods can provide valuable information for health interventions since they offer further in-depth knowledge and perspectives regarding the experience of resiliency to climate and air pollution stressors for different populations.

### 4.2. One Size Does Not Fit All: Contextual Factors Influencing Older Adult Health Risk

Contextual variation is critical in understanding the distribution of adverse health impacts due to climate- and air pollution-related exposures. The effects on older adult health in Canada depend on the intersection of multiple factors. Shin et al. reported higher risks of circulatory mortality from O_3_ in females over 65 years of age than males and younger females [61]. Additionally, comorbidities and low SES in older people often increased the risk of adverse health events. Region of residence also affected the vulnerability of older adults. A study in British Columbia found that older adults living in coastal areas of higher population density were more susceptible to adverse effects of extreme heat exposure, while in inland regions with lower population densities, very few effects varied by age [34]. The authors also suggested that further understanding of the mechanisms underlying varying sex-based health impacts would be essential [45]. Resilience to climate stressors does not depend on a single or even a few factors alone but complex interactions of factors. Improving our understanding of how contextual factors interact to reduce or promote resilience through exposure and sensitivity, spanning from the individual to the societal level, will enable effective strategies to meet the needs of older adults in our changing climate.

Fewer studies focused on social and behavioral factors that promote or reduce resilience to climatic and air pollution exposures in older adults; despite that, they are important determinants to consider. For example, different time–activity patterns between age strata may lead to differential exposures [30,45,59]. While this may influence our ability to detect age differences, it may also point to existing resiliency in these populations in Canada. In addition, Laverdière et al. identified that high social participation in older adults was strongly protective concerning adverse heat-related events. At the same time, low autonomy and poor health status were risk factors [44]. Promoting social participation among older adults through age-friendly cities, which aim to foster community engagement and active living environments, could be an effective strategy to reach isolated older people and prevent adverse health outcomes. Further qualitative and quantitative studies can refine our knowledge of social and behavioral indicators of risk and resiliency in older populations will help build resilience and promote the health of these people.

### 4.3. Missing Knowledge of the Health Risk Facing Immigrant Communities in Canada

In 2016, 21.9% of the Canadian population identified themselves as foreign born [13]. Yet, there was very little literature concerning climate- and air pollution-related health impacts in immigrant populations in Canada available, reflecting a critical knowledge gap in the field. Furthermore, there was none reporting on the impacts on older immigrants.

Immigrants in Canada are a heterogeneous group, and it is important to consider this diversity and differentiate risk and drivers of risk and resilience across sub-groups in future work. Immigrant populations may face barriers linked to language, socioeconomic status, access to healthcare, and have tendencies to settle in urban centers, which could affect resilience in these communities. The *healthy immigrant effect* diminishes with time, and older immigrants have more chronic conditions, which could exacerbate health impacts related to climate and air pollution [19,20,22]. Some research suggests that older immigrant women’s healthcare needs and concerns, specifically, are not being met by the healthcare system [86]. Moreover, immigrants who are racialized face different challenges than those who are non-racialized. For example, in those of prime work age (25–54 years), racialized immigrant men and women earn 71 and 79 cents, respectively, for one dollar that non-racialized immigrant men and women earn [87]. It will be important to consider these diverse experiences in future work to understand the drivers of health risk in immigrant populations.

### 4.4. Geographical Gaps in the Literature

There are substantial gaps in terms of the location of populations that have been studied. The impact of climatic and air pollution variables on health was studied most frequently in southern Ontario and Quebec, where the majority of the Canadian population resides. Several studies also focused on residents of British Colombia, mostly in urban centers. The Prairie and Maritimes provinces were largely understudied, with urban populations in Alberta being studied the most frequently within this group. No studies in the Territories were found, despite the fact that these northern regions are facing greater impacts from climate change [3]. The distribution in geographical coverage could be influenced by variation in air pollution concentrations across Canada. Generally, urban areas in southern Ontario, Alberta, and British Columbia experience higher levels of PM_2.5_, O_3_, and NO_2_ relative other regions [6]. There are also regions in British Columbia and Alberta that experience greater PM_2.5_ exposure in areas where summer wildfires are more frequent [6].

Variations in climate adaptation and mitigation policy between regions may also reflect research priorities and provide some explanation for geographical gaps. The Prairie Provinces, for example, have little to no climate policy in place and have reported increased emissions from 2005 to 2019 compared to Ontario and Quebec, where there is at least some leadership in climate policy and have reported decreased emissions [88].

Most publications were also set in urban areas, though some province-wide population studies included urban and rural residence indicators. Generally, those living in urban areas were at greater risk for air pollution and extreme temperatures, which is consistent with other work highlighting the Urban Heat Island (UHI) effect [88]. The UHI describes the higher average temperatures experienced by urban areas relative to nearby non-urban areas resulting from several factors, including density, land-use and travel proximity, and decreased green spaces. However, the health impacts of climate change in rural populations and the specific factors that influence this relationship in Canada are largely undetermined for older adults and immigrants.

Climate impacts can differ depending on the provincial and territorial weather patterns and geographies and provincial policies. At the same time, there are significant variations in contextual factors within Canada in terms of regional policy, population densities, demographics, and community characteristics. Capturing some of these important contextual factors for populations living across Canada can help identify where and which people are at greater risk for poorer health outcomes. Consequently, these understudied regions represent an important research gap in Canadian environmental health literature and warrant further investigation.

### 4.5. Limitations

There are limitations in this scoping review. Although the search strategy was intended to be comprehensive and included an extensive range of keywords, some papers may have been missed if the titles or keywords did not correspond with search parameters. In some cases, there were few studies on certain outcomes, which prevented a true synthesis. We also did not endeavor to assess the quality of evidence included in this review. The heterogeneity of exposure assessment, study design, and large number of health outcomes limits our ability to draw conclusions about the level of risk older adult’s face with respect to climatic and air pollution exposures. We also focused on a limited geographic area, limiting the generalizability of our findings to Canada.

## 5. Conclusions and Next Steps

The literature studied for this review indicated that older people living in Canada experience increased health risks due to climate- and air pollution-related exposures, and are influenced by other intersecting determinants. However, further studies are required to elucidate how these exposures intersect among themselves and other participating determinants to promote or reduce resiliency in this population. As little currently exists, more research about the health impacts of climate change and air pollution on immigrant populations is necessary, in addition to further studies on the health impacts on older adults and immigrants in understudied Canadian geographies, including the Prairies, Maritimes, and northern territories. The next steps could also involve investigating other populations in Canada facing greater risk to climate change and air pollution.

## Figures and Tables

**Table 1:** Climatic and air pollution variables and links to health in older adults and immigrant populations.

Authors	Population (*n*, age)	Primary Exposure Variables	Health Impact Category	Main Findings
Meteorological				
Vanasse 2017 [29]	112,793 participants; ≥65 years	Mean temperature	Cardiovascular	Decreasing temperature associated with increased risk in ≥65 years.
Bai 2018a [30]	~13 million participants; <65 years vs. ≥65 years	Mean, maximum, and extreme temperatures	Cardiovascular	Hot days associated with increased risk in ≥65 years.
Vida 2012 [31]	347,552 events; <65 years, ≥65 years	Mean temperature, diurnal temperature change	Mental health	Higher temperatures associated with increased risk in ≥65 years in an urban region.
Wang 2014 [32]	271,746 events; 0–14 years, 15–39 years, 50–59 years, ≥60 years	Minimum, maximum, mean temperature	Mental health	High temperatures associated with increased risk in ≥60 years.
Benmarhnia 2017 [33]	n NR <65 years vs. ≥65 years	Mean temperature	Mortality	Higher temperature associated with increased in ≥65 years.
Henderson 2013 [34]	~4.6 million participants; <75 years vs. ≥75 years	Apparent maximum temperature (°C)	Mortality	Higher temperature associated with increased mortality in ≥75 years living in the coastal region.
Vutcovici 2014 [35]	Mean 30.1 (6.9 SD) deaths/day for 8766 days; ≥65 years	Diurnal temperature change	Mortality	Increased diurnal temperature change associated with mortality.
Kosatsky 2012 [36]	398 participants; <65 years, 65–75 years, 75–85 years, ≥85	Heatwave	Mortality; cardiovascular; respiratory	Heatwaves associated with increased risk in 65–74 years.
Chen 2016 [37]	352,818 participants; <65 years vs. ≥65 years	Mean temperature	Mortality; cardiovascular; respiratory; diabetes	Cold temperatures associated with increased cardiovascular mortality in ≥65 years.
Bustinza 2013 [38]	~6 million people; <65 years, 65–74 years, ≥75 years	Heatwave	Mortality; overall morbidity	Signficant increase in crude death rates for ≥75 years.
Burton 2015 [39]	NR	Flooding	Mortality; respiratory; mental health	Older adults at risk of adverse health events due to flooding.
Mondor 2015 [40]	136,323 participants; ≥65 years	Freezing rain, snowstorms	Fall-related injuries	Freezing rain associated with increased risk in ≥65 years.
Modarres 2012 [41]	1,077,813 participants; 40–74 years, ≥75 years	Minimum, maximum, mean temperature, precipitation, snow depth, daylight hours, air pressure	Fall-related injuries	Decreasing temperature, rainfall, daylight hours, and increased snow associated with increased risk in ≥75 years.
Modarres 2014 [42]	~900,000 participants; 40–74 years, ≥75 years	Minimum, maximum, mean temperature, precipitation, snow depth, daylight hours, air pressure	Fall-related injuries	Maximum pressure and daylight hours associated with increased risk older adults. Rainfall depth was a predictor for older males.
Auger 2017 [43]	14,302 participants; <55 years, 55–64 years, 65–74 years, ≥75 years	Mean temperature	Ocular	Increased temperature associated with increased risk in 65–74 years.
Laverdière 2016 [44]	1233 participants; 68–72 years, 73–77 years, 78–82 years	Daily maximum temperature ≥ 30 °C	Overall morbidity	Hot days associated with increased risk.
Bélanger 2014 * [45]	3485 participants; 18–35 years, 35–44 years, 45–54 years, 55–64 years, ≥65 years	Hot and humid days	Overall morbidity	Increased prevalence in low income ≥ 65 years, decreased prevalence in foreign born residents.
Stapleton 2014 [46]	24 participants; 12 younger participants, mean 21 years (3 SD); 12 older participants, mean 65 years (5 SD)	Hot-dry and hot-humid conditions	Overall morbidity	Hot conditions associated with a significant increase in body heat content in older adults compared to younger.
Tajmir 2013 [47]	18 participants 10 younger; mean 26 (2.4 SD) years; 8 older; mean 68 (4.4 SD) years	Temperature categories; 23 °C vs. 1 °C	Overall morbidity	Cold conditions associated with greater impairment of manual movements in and reduced sensitivity.
McTavish 2018 [48]	227,135 participants Median 80 (74–85 IQR) years	Maximum temperature	Renal	High heat marginally associated with greater risk of acute kidney injury.
Ordon 2015 [49]	423,396 participants; 18–39 years, 40–49 years, 50–59 years, 60–69 years, >70 years	Minimum, maximum, mean temperature, extreme temperatures	Renal	Extreme heat associated with increased risk in 60–69 years.
Meteorological and Air pollution				
Krstic 2011 [50]	≥65 years	Steadman’s apparent temperature, PM_2.5_	Mortality; cardiovascular; respiratory	High and low temperatures associated with increased risk.
Air pollution				
Parent 2013 * [51]	1772 participants; <60 years, 60–66 years, 67–71 years, >71 years	NO_2_	Cancer	Increased risk in third quartile distribution of recent immigrants.
Goldberg 2018 [52]	788 participants; 50–70 years	UFP	Cancer	Positive but insignificant associations.
Goldberg 2017 [53]	1277 participants; Cases mean 62.23 years (SD NR), controls 61.03 years (SD NR)	NO_2_, UFP	Cancer	Positive but insignificant associations.
Chen 2014 [54]	5229 participants; 25–44 years, 45–64 years, 65–74 years, 75–84 years, ≥85 years	AQHI, CO, NO_2_, O_3_, PM_2.5,_ PM_10,_ SO_2_	Cardiovascular	AQHI, CO, and NO_2_ associated with increased risk in ≥75 years.
Wang 2015 [55]	25,894 participants; <65 years vs. ≥65 years	CO, NO_2_, NO, O_3_, PM_2.5_	Cardiovascular	Observed positive associations with NO_2_ and NO and negative associations with CO in ≥65 years.
Weichenthal 2017 [56]	2881 events; <65 years vs. ≥65 years	PM_2.5_, NO_2_, O_3_	Cardiovascular	PM_2.5_ associated with increased risk in ≥65 years.
Gan 2011 [57]	452,735 participants; <60 years, 60–69 years, ≥70 years	BC, NO, NO_2_, PM_2.5_	Cardiovascular	BC associated with increased risk of mortality in ≥60 years and hospitalizations in 60–69 years.
Shin 2019 [58]	5,071,956 participants; 35–44 years, 45–54 years, 55–64 years, 65–74 years, 75–85 years	NO_2_, O_3_, O_x_, PM_2.5_	Cardiovascular	All pollutants were associated with increased risk of cardiovascular outcomes.
Bai 2019 [59]	50,062,146 and 5,141,172 participants; 35–44 years, 45–54 years, 55–64 years, 65–74 years, 75–85 years;	PM_2.5_, NO_2_, O_3_, O_x_	Cardiovascular	All pollutants associated with increased risk in ≥65 years.
Shin 2018a [60]	2,194,519 participants; <50 years, ≥50 years, ≥65 years	O_3_	Cardiovascular	No significant associations ≥ 50 years.
Shin 2020 [61]	~19 million participants; 1–65 years vs. >65 years	O_3_	Cardiovascular	O_3_ associated with increased risk in ≥65 years.
Stieb 2017 [62]	2013: 36 participants, 2014: 41 participants; 55–59 years, 60–64 years, 65–69 years, ≥70 years	AQHI, CO, NO_2_, O_3_, PM_2.5_, SO_2_	Cardiovascular	AQHI, PM_2.5_, and O_3_ associated with subclinical adverse cardio-respiratory effects.
Stieb 2018 [63]	2014: 36 participants, 2015: 34 participants; 55–59 years, 60–64 years, 65–69 years, ≥70 years	AQHI, CO, NO_2_, O_3_, O_x_, PM_2.5_, SO_2_	Cardiovascular	AQHI, PM_2.5_, O_3_ and O_x_ associated with subclinical cardio-respiratory effects.
Stieb 2019 [64]	72 participants; 55–81 years	AQHI, CO, NO_2_, O_3_, PM_2.5_	Cardiovascular	AQHI and PM_2.5_ associated with subclinical cardio-respiratory effects and markers of oxidative stress.
Bai 2018b [65]	Hypertension: 893,499 participants; Mean 48.6 (SD 14.3) years; Diabetes: 1,056,012 participants; Mean 51.1 (15.3 SD) years	UFP, NO_2_	Cardiovascular; diabetes	UFP and NO_2_ associated with diabetes and hypertension; NO_2_ negatively associated with hypertension in 60–74 years.
Henderson 2011 [66]	281,711 participants; 0–5 years, 5–10 years, 10–20 years, 20–30 years, 30–40 years, 40–50 years, 50–60 years, 60–70 years, 70–80 years, ≥80 years	PM_10_	Cardiovascular; respiratory	PM_10_ was associated with increased risk in 60–70 years and ≥80 years.
Crouse 2015 [67]	735,590 participants; 25–34 years, 35–44 years, 45–54 years, 55–64 years, 65–74 years, ≥75 years	NO_2_	Cardiovascular; respiratory; diabetes; mortality	NO_2_ associated with increased risk in 60–79 years.
Farhat 2013 [68]	~9.1 million participants; <75 years vs. ≥75 years	O_3_, PM_2.5_	Cardiovascular; respiratory; mortality	O_3_ and PM_2.5_ were associated with increased risk in those ≥75 years.
Goldberg 2013 [69]	158,350 participants; ≥65 years	CO, NO_2_, O_3_, PM_2.5_, SO_2_	Mortality; cancer; cardiovascular; respiratory; diabetes	All pollutants associated with increased risk.
Vanos 2013 [70]	n NR <65 years, 65–74 years, 75–84 years, ≥85 years	CO, NO_2_, O_3_, SO_2_	Mortality	All pollutants associated with increased risk in ≥85 years.
de Roos 2014 [71]	678,361 participants; <65 years vs. ≥65 years	Proximity to roads, BC, CO, NO, NO_2_, O_3_, PM_2.5_, PM_10_	Musculo-skeletal	O_3_ associated with increased risk in ≥65 years.
Chen 2017a [72]	2,066,639 participants; 55–64 years, 65–74 years, 75–85 years	NO_2_, O_3_, PM_2.5_	Neurological	PM_2.5_ and NO_2_ associated with increased risk of dementia in those 55–85 years.
Chen 2017b [73]	2,165,269 participants; Mean 66.8 (8.2 SD) years	NO_2,_ Proximity to roads, PM_2.5_	Neurological	Closer proximity to roads, PM_2.5_ and NO_2_ associated with increased risk of dementia, PM_2.5_ associated with increased risk of Parkinson’s.
Shin 2018b [74]	2,194,519 participants; 55–85 years	NO_2_, O_3_, PM_2.5_	Neurological	PM_2.5_ and O_3_ associated with increased risk.
Neupane 2010 [75]	859 participants; ≥65 years	NO_2_, PM_2.5_, SO_2_	Respiratory	NO_2_ and PM_2.5_ were associated with increased risk.
Szyszkowicz 2014 [76]	6697 participants; 2–14 years, 15–39 years, 40–59 years, ≥60 years	AQHI, NO_2_, O_3_, PM_2.5_	Respiratory	No significant associations in ≥60 years observed.
Lavigne 2012 [77]	3728 participants; 2–14 years, 15–39 years, 40–59 years, ≥60 years	NO_2_, CO, PM_2.5_, SO_2_	Respiratory	O_3_ associated with increased risk during cold season in ≥60 years.
Gan 2013 [78]	467,994 participants; Mean 60 (11 SD) years	BC, NO, NO_2_, PM_2.5_	Respiratory	BC associated with increased risk in ≥65 years.
To 2013 [79]	~1.5 million participants; 0–4 years, 5–9 years, 10–19 years, 20–59 years, ≥60 years	AQHI, NO_2_, O_3_, PM_2.5_	Respiratory	AQHI was associated with asthma in ≥60 years.
Ward 2015 [80]	107,108 participants; 0–19 years, 20–64 years, ≥65 years	AQI, CO, O_3_, PM_2.5_	Respiratory	No significant associations observed in ≥65 years.

* Reported results specific to immigrant populations. AQHI—Air Quality Health Index; AQI—Air Quality Index; BC— black carbon; UFP—ultrafine particles (≤0.1 µm diameter).

**Table 2:** Summary of outcomes and associated meteorological parameters in older adults. An increase or decrease in the value of a parameter is denoted by ↑ or ↓, respectively.

Outcome Category	Climate-Related Exposures
Mortality	↑ Temperature Diurnal temperature variation Heatwave Flooding
Overall morbidity	↑ Temperature Heat waves
Cardiovascular	↑ Temperature Extreme heat Extreme cold Heatwave
Respiratory	↑ Temperature Heatwave Flooding
Fall-related injuries	↓ Temperature Precipitation Snow depth Daylight hours Air pressure
Mental health	↑ Temperature ↑Humidity Heatwave Flooding
Renal	↑ Temperature Heatwave
Ocular health	↑ Temperature Heatwave
Diabetes	Extreme heat *(null)* Extreme cold *(null)*

**Table 3:** Summary of outcomes and associated air pollution exposures in older adults.

Outcome Category	Air Pollution Exposures
Cardiovascular	AQHI, AQI, BC, CO, NO, NO_2_, O_3_, O_x_, PM_2.5_, PM_10_, SO_2_, UFP
Respiratory	AQHI, BC, CO, NO, NO_2_, O_3_, PM_2.5_, PM_10_, SO_2_, UFP
Cancer	CO, NO_2_, O_3_, PM_2.5_, SO_2_, UFP
Neurological	NO_2_, O_3_, PM_2.5_
Diabetes	CO, NO_2_, O_3_, PM_2.5_, SO_2_
Mortality	CO, NO_2_, O_3_, PM_10_, SO_2_
Musculoskeletal outcomes	Proximity to roads, BC, CO, NO, NO_2_, O_3_, PM_2.5_, PM_10_

## Data Availability

No new data were created or analyzed in this study. Data sharing is not applicable to this article.

## Supplementary Materials

The following are available online at https://www.mdpi.com/article/10.3390/ijerph182010575/s1, Table S1. Detailed search string. Table S2. Climatic and air pollution variables and links to health in older adults and immigrant populations.

## References

[1] World Health Organization (2015). Did You Know? By Taking Action on Climate Change You Can Strengthen Public Health.

[2] World Health Organization (2015). Health and the Environment: Addressing the Health Impact of Air Pollution.

[3] Bush E., Lemmen D.S. (2019). Canada’s Changing Climate Report.

[4] Smith K.R., Woodward A., Campbell-Lendrum D., Chadee D.D., Honda Y., Qiyong L., Olwoch J.M., Revich B., Sauerborn R. (2015). Human health: Impacts, adaptation, and co-benefits. Climate Change 2014 Impacts, Adaptation and Vulnerability: Part A: Global and Sectoral Aspects.

[5] Warren F.J., Lemmen D.S. (2014). Canada in a Changing Climate: Sector Perspectives on Impacts and Adaptation.

[6] Health Canada (2021). Health Impacts of Air Pollution in Canada Estimates of Premature Deaths and Nonfatal Outcomes.

[7] Alberini A., Gans W., Alhassan M. (2011). Individual and Public-Program Adaptation: Coping with Heat Waves in Five Cities in Canada. Int. J. Environ. Res. Public Health.

[8] Manangan A.P., Uejio C.K., Saha S., Schramm P.J., Marinucci G.D., Brown C.L., Hess J.J., Luber G. (2016). Assessing health vulnerability to climate change: A guide for health departments. Climate Change and Public Health: Federal Preparedness Efforts.

[9] Benevolenza M.A., DeRigne L. (2019). The impact of climate change and natural disasters on vulnerable populations: A systematic review of literature. J. Hum. Behav. Soc. Environ..

[10] Seguin J. (2008). Human Health in a Changing Climate: A Canadian Assessment of Vulnerabilities and Adaptive Capacity. Human Health in a Changing Climate.

[11] Statistics Canada (2010). Population Projections for Canada.

[12] Morency J.-D., Caron-Malenfant E., MacIsaac S., Statistics Canada (2017). Immigration and diversity: Population projections for Can-ada and its regions, 2011 to 2036.

[13] Statistics Canada (2017). Immigration and Ethnocultural Diversity: Key Results from the 2016 Census.

[14] Åström C., Orru H., Rocklöv J., Strandberg G., Ebi K.L., Forsberg B. (2013). Heat-related respiratory hospital admissions in Europe in a changing climate: A health impact assessment. BMJ Open.

[15] Abrahamson V., Wolf J., Lorenzoni I., Fenn B., Kovats S., Wilkinson P., Adger W.N., Raine R. (2008). Perceptions of heatwave risks to health: Interview-based study of older people in London and Norwich, UK. J. Public Health.

[16] Kenny G.P., Yardley J., Brown C., Sigal R.J., Jay O. (2010). Heat stress in older individuals and patients with common chronic diseases. Can. Med. Assoc. J..

[17] Bunker A., Wildenhain J., Vandenbergh A., Henschke N., Rocklöv J., Hajat S., Sauerborn R. (2016). Effects of Air Temperature on Climate-Sensitive Mortality and Morbidity Outcomes in the Elderly; a Systematic Review and Meta-analysis of Epidemiological Evidence. EBioMedicine.

[18] Leyva E.W.A., Beaman A., Davidson P.M. (2017). Health Impact of Climate Change in Older People: An Integrative Review and Implications for Nursing. J. Nurs. Sch..

[19] Vang Z.M., Sigouin J., Flenon A., Gagnon A. (2017). Are immigrants healthier than native-born Canadians? A systematic review of the healthy immigrant effect in Canada. Ethn. Health.

[20] Beiser M. (2005). The Health of Immigrants and Refugees in Canada. Can. J. Public Health.

[21] Hansen A., Bi L., Saniotis A., Nitschke M. (2013). Vulnerability to extreme heat and climate change: Is ethnicity a factor?. Glob. Health Action.

[22] McMichael C., Barnett J., McMichael A.J. (2012). An Ill Wind? Climate Change, Migration, and Health. Environ. Health Perspect..

[23] Otto I.M., Reckien D., Reyer C., Marcus R., Le Masson V., Jones L., Norton A., Serdeczny O. (2017). Social vulnerability to climate change: A review of concepts and evidence. Reg. Environ. Chang..

[24] Tilstra M.H., Tiwari I., Niwa L., Campbell S., Jones C.A., Quemerais B., Yamamoto S.S. (2020). Characterising Sensitivity to Climate Change in Older Adults and Immigrants in Canada—A Scoping Review Protocol. Open Science Framework Web. OSF.

[25] Arksey H., O’Malley L. (2005). Scoping studies: Towards a methodological framework. Int. J. Soc. Res. Methodol..

[26] Tricco A.C., Lillie E., Zarin W., O’Brien K.K., Colquhoun H., Levac D., Moher D., Peters M., Horsley T., Weeks L. (2018). PRISMA Extension for Scoping Reviews (PRISMA-ScR): Checklist and Explanation. Ann. Intern. Med..

[27] International Health Conference (1946). Constitution of the World Health Organization. Public Health Rep..

[28] Statistics Canada Immigrant n.d. https://www23.statcan.gc.ca/imdb/p3Var.pl?Function=Unit&Id=85107.

[29] Vanasse A., Talbot D., Chebana F., Bélanger D., Blais C., Gamache P., Giroux J.-X., Dault R., Gosselin P. (2017). Effects of climate and fine particulate matter on hospitalizations and deaths for heart failure in elderly: A population-based cohort study. Environ. Int..

[30] Bai L., Li Q., Wang J., Lavigne E., Gasparrini A., Copes R., Yagouti A., Burnett R.T., Goldberg M.S., Cakmak S. (2018). Increased coronary heart disease and stroke hospitalisations from ambient temperatures in Ontario. Hearth.

[31] Vida S., Durocher M., Ouarda T.B.M.J., Gosselin P. (2012). Relationship Between Ambient Temperature and Humidity and Visits to Mental Health Emergency Departments in Québec. Psychiatr. Serv..

[32] Wang X., Lavigne E., Ouellette-Kuntz H., Chen B.E. (2014). Acute impacts of extreme temperature exposure on emergency room admissions related to mental and behavior disorders in Toronto, Canada. J. Affect. Disord..

[33] Benmarhnia T., Kaufman J.S. (2017). When evidence of heat-related vulnerability depends on the contrast measure. Int. J. Biometeorol..

[34] Henderson S.B., Wan V., Kosatsky T. (2013). Differences in heat-related mortality across four ecological regions with diverse urban, rural, and remote populations in British Columbia, Canada. Health Place.

[35] Vutcovici M., Goldberg M.S., Valois M.-F. (2014). Effects of diurnal variations in temperature on non-accidental mortality among the elderly population of Montreal, Québec, 1984–2007. Int. J. Biometeorol..

[36] Kosatsky T., Henderson S.B., Pollock S.L. (2012). Shifts in Mortality During a Hot Weather Event in Vancouver, British Columbia: Rapid Assessment With Case-Only Analysis. Am. J. Public Health.

[37] Chen H., Wang J., Li Q., Yagouti A., Lavigne E., Foty R., Burnett R.T., Villeneuve P.J., Cakmak S., Copes R. (2016). Assessment of the effect of cold and hot temperatures on mortality in Ontario, Canada: A population-based study. CMAJ Open.

[38] Bustinza R., Lebel G., Gosselin P., Bélanger D., Chebana F. (2013). Health impacts of the July 2010 heat wave in Québec, Canada. BMC Public Health.

[39] Burton H., Rabito F., Danielson L., Takaro T.K. (2016). Health effects of flooding in Canada: A 2015 review and description of gaps in research. Can. Water Resour. J..

[40] Mondor L., Charland K., Verma A., Buckeridge D.L. (2015). Weather warnings predict fall-related injuries among older adults. Age Ageing.

[41] Modarres R., Ouarda T.B., Vanasse A., Orzanco M.G., Gosselin P. (2012). Modeling seasonal variation of hip fracture in Montreal, Canada. Bone.

[42] Modarres R., Ouarda T.B.M.J., Vanasse A., Orzanco M.G., Gosselin P. (2014). Modeling climate effects on hip fracture rate by the multivariate GARCH model in Montreal region, Canada. Int. J. Biometeorol..

[43] Auger N., Rhéaume M.-A., Bilodeau-Bertrand M., Tang T., Kosatsky T. (2017). Climate and the eye: Case-crossover analysis of retinal detachment after exposure to ambient heat. Environ. Res..

[44] Laverdière É., Payette H., Gaudreau P., Morais J.A., Shatenstein B., Généreux M. (2016). Risk and protective factors for heat-related events among older adults of Southern Quebec (Canada): The NuAge study. Can. J. Public Health.

[45] Bélanger D., Gosselin P., Valois P., Abdous B. (2014). Perceived Adverse Health Effects of Heat and Their Determinants in Deprived Neighbourhoods: A Cross-Sectional Survey of Nine Cities in Canada. Int. J. Environ. Res. Public Health.

[46] Stapleton J.M., LaRose J., Simpson C., Flouris A., Sigal R.J., Kenny G.P. (2014). Do older adults experience greater thermal strain during heat waves?. Appl. Physiol. Nutr. Metab..

[47] Tajmir P., Grierson L.E.M., Carnahan H. (2013). Interactions between Cold Ambient Temperature and Older Age on Haptic Acuity and Manual Performance. Can. J. Aging.

[48] McTavish R.K., Richard L., McArthur E., Shariff S.Z., Acedillo R., Parikh C.R., Wald R., Wilk P., Garg A.X. (2018). Association Between High Environmental Heat and Risk of Acute Kidney Injury Among Older Adults in a Northern Climate: A Matched Case-Control Study. Am. J. Kidney Dis..

[49] Ordon M., Welk B., Li Q., Wang J., Lavigne E., Yagouti A., Copes R., Cakmak S., Chen H. (2016). Ambient Temperature and the Risk of Renal Colic: A Population-Based Study of the Impact of Demographics and Comorbidity. J. Endourol..

[50] Krstić G. (2011). Apparent Temperature and Air Pollution vs. Elderly Population Mortality in Metro Vancouver. PLoS ONE.

[51] Parent M.-É., Goldberg M.S., Crouse D., Ross N.A., Chen H., Valois M.-F., Liautaud A. (2013). Traffic-related air pollution and prostate cancer risk: A case–control study in Montreal, Canada. Occup. Environ. Med..

[52] Goldberg M.S., Labrèche F., Weichenthal S., Lavigne E., Valois M.-F., Hatzopoulou M., Shekarrizfard M. (2018). Number concentrations of ultrafine particles and the incidence of postmenopausal breast cancer. Environ. Epidemiol..

[53] Goldberg M.S., Labrèche F., Weichenthal S., Lavigne E., Valois M.-F., Hatzopoulou M., Van Ryswyk K., Shekarrizfard M., Villeneuve P., Crouse D. (2017). The association between the incidence of postmenopausal breast cancer and concentrations at street-level of nitrogen dioxide and ultrafine particles. Environ. Res..

[54] Chen L., Villeneuve P.J., Rowe B.H., Liu L., Stieb D.M. (2014). The Air Quality Health Index as a predictor of emergency department visits for ischemic stroke in Edmonton, Canada. J. Expo. Sci. Environ. Epidemiol..

[55] Wang X., Kindzierski W.B., Kaul P. (2015). Air Pollution and Acute Myocardial Infarction Hospital Admission in Alberta, Canada: A Three-Step Procedure Case-Crossover Study. PLoS ONE.

[56] Weichenthal S., Kulka R., Lavigne E., van Rijswijk D., Brauer M., Villeneuve P.J., Stieb D., Joseph L., Burnett R.T. (2017). Biomass Burning as a Source of Ambient Fine Particulate Air Pollution and Acute Myocardial Infarction. Epidemiology.

[57] Gan W.Q., Koehoorn M., Davies H.W., Demers P.A., Tamburic L., Brauer M. (2011). Long-Term Exposure to Traffic-Related Air Pollution and the Risk of Coronary Heart Disease Hospitalization and Mortality. Environ. Health Perspect..

[58] Shin S., Burnett R.T., Kwong J.C., Hystad P., Van Donkelaar A., Brook J.R., Goldberg M.S., Tu K., Copes R., Martin R.V. (2019). Ambient Air Pollution and the Risk of Atrial Fibrillation and Stroke: A Population-Based Cohort Study. Environ. Health Perspect..

[59] Bai L., Shin S., Burnett R.T., Kwong J.C., Hystad P., Van Donkelaar A., Goldberg M.S., Lavigne E., Copes R., Martin R.V. (2019). Exposure to ambient air pollution and the incidence of congestive heart failure and acute myocardial infarction: A population-based study of 5.1 million Canadian adults living in Ontario. Environ. Int..

[60] Shin H.H., Burr W.S., Stieb D., Haque L., Kalayci H., Jovic B., Smith-Doiron M. (2018). Air health trend indicator: Association between short-term exposure to ground ozone and circulatory hospitalizations in Canada for 17 years, 1996–2012. Int. J. Environ. Res. Public Health..

[61] Shin H.H., Parajuli R.P., Maquiling A., Smith-Doiron M. (2020). Temporal trends in associations between ozone and circulatory mortality in age and sex in Canada during 1984–2012. Sci. Total Environ..

[62] Stieb D.M., Shutt R., Kauri L., Mason S., Chen L., Szyszkowicz M., Dobbin N.A., Rigden M., Jovic B., Mulholland M. (2017). Cardio-Respiratory Effects of Air Pollution in a Panel Study of Outdoor Physical Activity and Health in Rural Older Adults. J. Occup. Environ. Med..

[63] Stieb D.M., Shutt R., Kauri L., Roth G., Szyszkowicz M., Dobbin N.A., Chen L., Rigden M., Van Ryswyk K., Kulka R. (2018). Cardiorespiratory Effects of Air Pollution in a Panel Study of Winter Outdoor Physical Activity in Older Adults. J. Occup. Environ. Med..

[64] Stieb D.M., Shutt R.H., Kauri L.M., Mason-Renton S., Chen L., Szyszkowicz M., Dobbin N.A., Rigden M., Jovic B., Mulholland M. (2019). Associations between air pollution and cardio-respiratory physiological measures in older adults exercising outdoors. Int. J. Environ. Health Res..

[65] Bai L., Chen H., Hatzopoulou M., Jerrett M., Kwong J.C., Burnett R.T., Van Donkelaar A., Copes R., Martin R.V., Van Ryswyk K. (2018). Exposure to Ambient Ultrafine Particles and Nitrogen Dioxide and Incident Hypertension and Diabetes. Epidemiology.

[66] Henderson S.B., Brauer M., Macnab Y.C., Kennedy S.M. (2011). Three Measures of Forest Fire Smoke Exposure and Their Associations with Respiratory and Cardiovascular Health Outcomes in a Population-Based Cohort. Environ. Health Perspect..

[67] Crouse D.L., Peters P.A., Villeneuve P., Proux M.-O., Shin H.H., Goldberg M.S., Johnson M., Wheeler A., Allen R.W., Atari D.O. (2015). Within- and between-city contrasts in nitrogen dioxide and mortality in 10 Canadian cities; a subset of the Canadian Census Health and Environment Cohort (CanCHEC). J. Expo. Sci. Environ. Epidemiol..

[68] Farhat N., Ramsay T., Jerrett M., Krewski D. (2013). Short-Term Effects of Ozone and PM2.5 on Mortality in 12 Canadian Cities. J. Environ. Prot..

[69] Goldberg M.S., Burnett R.T., Stieb D.M., Brophy J., Daskalopoulou S.S., Valois M.-F., Brook J.R. (2013). Associations between ambient air pollution and daily mortality among elderly persons in Montreal, Quebec. Sci. Total Environ..

[70] Vanos J.K., Cakmak S., Bristow C., Brion V., Tremblay N., Martin S.L., Sheridan S.S. (2013). Synoptic weather typing applied to air pollution mortality among the elderly in 10 Canadian cities. Environ. Res..

[71] De Roos A.J., Koehoorn M., Tamburic L., Davies H.W., Brauer M. (2014). Proximity to Traffic, Ambient Air Pollution, and Community Noise in Relation to Incident Rheumatoid Arthritis. Environ. Health Perspect..

[72] Chen H., Kwong J.C., Copes R., Hystad P., van Donkelaar A., Tu K., Brook J.R., Goldberg M.S., Martin R.V., Murray B. (2017). Exposure to ambient air pollution and the incidence of dementia: A population-based cohort study. Environ. Int..

[73] Chen H., Kwong J.C., Copes R., Tu K., Villeneuve P., van Donkelaar A., Hystad P., Martin R.V., Murray B., Jessiman B. (2017). Living near major roads and the incidence of dementia, Parkinson’s disease, and multiple sclerosis: A population-based cohort study. Lancet.

[74] Shin S., Burnett R.T., Kwong J.C., Hystad P., Van Donkelaar A., Brook J.R., Copes R., Tu K., Goldberg M.S., Villeneuve P. (2018). Effects of ambient air pollution on incident Parkinson’s disease in Ontario, 2001 to 2013: A population-based cohort study. Int. J. Epidemiology.

[75] Neupane B., Jerrett M., Burnett R.T., Marrie T., Arain M.A., Loeb M. (2010). Long-Term Exposure to Ambient Air Pollution and Risk of Hospitalization with Community-acquired Pneumonia in Older Adults. Am. J. Respir. Crit. Care Med..

[76] Szyszkowicz M., Kousha T. (2014). Emergency department visits for asthma in relation to the Air Quality Health Index: A case-crossover study in Windsor, Canada. Can. J. Public Health.

[77] Lavigne E., Villeneuve P., Cakmak S. (2012). Air Pollution and Emergency Department Visits for Asthma in Windsor, Canada. Can. J. Public Health.

[78] Gan W.Q., Fitzgerald J.M., Carlsten C., Sadatsafavi M., Brauer M. (2013). Associations of Ambient Air Pollution with Chronic Obstructive Pulmonary Disease Hospitalization and Mortality. Am. J. Respir. Crit. Care Med..

[79] To T., Shen S., Atenafu E., Guan J., McLimont S., Stocks B., Licskai C. (2013). The Air Quality Health Index and Asthma Morbidity: A Population-Based Study. Environ. Health Perspect..

[80] Ward C.J. (2015). It’s an ill wind: The effect of fine particulate air pollution on respiratory hospitalizations. Can. J. Econ..

[81] Cohen G., Gerber Y. (2017). Air Pollution and Successful Aging: Recent Evidence and New Perspectives. Curr. Environ. Health Rep..

[82] Arbuthnott K.G., Hajat S. (2017). The health effects of hotter summers and heat waves in the population of the United Kingdom: A review of the evidence. Environ. Health.

[83] Hajat S. (2017). Health effects of milder winters: A review of evidence from the United Kingdom. Environ. Health.

[84] Gamble J.L., Hurley B.J., Schultz P.A., Jaglom W.S., Krishnan N., Harris M. (2013). Climate Change and Older Americans: State of the Science. Environ. Health Perspect..

[85] Guruge S., Birpreet B., Samuels-Dennis J.A. (2015). Health Status and Health Determinants of Older Immigrant Women in Canada: A Scoping Review. J. Aging Res..

[86] Block S., Galabuzi G.-E., Tranjan R. (2019). Canada’s Colour Coded Income Inequality.

[87] Dusyk N., Turcotte I., Gunton T., Macnab J., Mcbain S., Penney N., Pickrell-Barr J., Pope M. (2021). All Hands on Deck: An Assessment of Provincial, Terri-Torial and Federal Readiness to Deliver a Safe Climate.

[88] Heaviside C., Macintyre H., Vardoulakis S. (2017). The Urban Heat Island: Implications for Health in a Changing Environment. Curr. Environ. Health Rep..

