# Supplementary material for: Risk and Resilience: How Is the Health of Older Adults and Immigrant People Living in Canada Impacted by Climate- and Air Pollution-Related Exposures?
Source: Int J Environ Res Public Health. 2021 Oct 9;18(20):10575. doi: 10.3390/ijerph182010575 (PMC8535805; doi:10.3390/ijerph182010575)
Supplement: Supplementary file 1 [file ijerph-18-10575-s001.zip › Tilstra_IJERPH_S1.pdf]

Supplementary Materials

Table S1. Detailed search strings

Ovid MEDLINE(R) ALL <1946 to May 22, 2020>

#	Search Statement	Results
1	weather/ or Climate/ or climatic processes/ or ((exp climate change/ or cyclonic storms/ or droughts/ or floods/ or greenhouse effect/ or temperature/) and (precipitation or heat or cold or ambient or season* or climate or climatic or weather or summer or winter).ti,ab,kw.) or extreme heat/ or hot temperature/ or (((climat* not (political-climate or organizational-climate or economic-climate or financial-climate)) or weather) adj3 (chang* or disrupt* or volati* or instabilit* or unstable or variable or variability or vulnerab*)).ti,ab,kw. or ((chang* or decline* or decrease* or increase*) adj3 humidity).ti,ab,kw. or ((global* or climate) adj2 warm*).ti,ab,kw. or ((climat* or weather) adj (conditions or factors or driven)).ti,ab,kw. or (greenhouse gas* or greenhouse effect* or carbon emission* or carbon dioxide emission* or CO2 emission*).ti,ab,kw. or (((extreme or severe) adj (weather or heat or temperature*)) or hot-weather or heat-wave* or heatwave or high temperatures or ((higher or warm* or hot*) adj3 temperature*)).ti,ab,kw. or ((annual* or daily or month* or ambient or environmental or season* or climate or climatic or weather or summer or winter) adj4 temperature*).ti,ab,kw. or (warm-season* or warm*-month* or (unseasonabl* adj3 (warm or hot or heat or humid* or cold or high or low))).ti,ab,kw. or (El Nino or la nina).ti,ab,kw. or (rain or rains or rainstorm* or raining or rainfall or ((high or heavy or extreme or severe or daily or levels) adj2 precipitation) or UV-index or flooding or floods or waterlogging or (drought* not drought-resistan*) or desertifcation or hurricane* or cyclone* or tornado* or superstorm* or dust storm or storm surge* or ice storm* or ((storm or storms) and disaster*) or monsoon*).ti,ab,kw. or (forest fire* or wildfire* or wild fire* or fire disaster* or (uncontrolled adj3 fire*)).ti,ab,kw. or ((sea level* adj3 (rise or rises or rising or increase or increasing or increases)) or ((polar or permafrost or ice-cap or glacier*) adj3 (melt* or retreat* or reced* or thaw*))).ti,ab,kw. or famine*.ti,ab,kw.	344496

2	exp Air Pollution/ or Traffic-Related Pollution/ or greenhouse gases/ or stratospheric ozone/ or ((gases/ or ammonia/ or carbon dioxide/ or carbon monoxide/ or chlorine/ or greenhouse gases/ or hydrogen sulfide/ or nitrogen/ or nitrogen oxides/ or nitrogen dioxide/ or nitrous oxide/ or ozone/ or sulfur dioxide/ or carbon monoxide*.ti,ab,kw. or hydrogen sulfide*.ti,ab,kw. or hydrogen sulphide*.ti,ab,kw. or H2S.ti,ab,kw. or nitrogen oxide*.ti,ab,kw. or volatile organic*.ti,ab,kw. or voc.ti,ab,kw. or nox.ti,ab,kw. or sulfur dioxide*.ti,ab,kw. or SO2.ti,ab,kw. or sulfur oxide*.ti,ab,kw. or sulphur dioxide*.ti,ab,kw. or sulphur oxide*.ti,ab,kw.) and (exp Pollution/ or pollut*.ti,ab,kw. or emit*.ti,ab,kw. or emission*.ti,ab,kw. or contaminat*.ti,ab,kw. or decontaminat*.ti,ab,kw.)) or ((air or atmospher*) adj3 (pollut* or quality or wast* or contamin* or emission* or immission* or effluent*or acidification or contaminat* or degrad* or decontaminat* or purif* or restor*)).ti,ab,kw. or ((gas or gases) adj3 (toxic* or noxious or releas* or purifi* or scrub* or emit* or waste* or vapo?r)).ti,ab,kw. or (acid* adj3 (deposition* or rain* or snow or fog or mist or precipitation or hail or sleet)).ti,ab,kw. or afterburning.ti,ab,kw. or biogas.ti,ab,kw. or (burning adj3 fossil fuel*).ti,ab,kw. or ((chimney or stack) adj3 height).ti,ab,kw. or dust.ti,ab,kw. or fallout.ti,ab,kw. or fall out.ti,ab,kw. or flaring.ti,ab,kw. or fly ash.ti,ab,kw. or electrosmog.ti,ab,kw. or (greenhouse adj3 (gas or effect or effects or gases or emission*)).ti,ab,kw. or incineration.ti,ab,kw. or odor.ti,ab,kw. or odour.ti,ab,kw. or olfactory pollution.ti,ab,kw. or particulate*.ti,ab,kw. or smog.ti,ab,kw. or smoke.ti,ab,kw. or soot.ti,ab,kw. or sick building syndrome.ti,ab,kw. or vapo?r recovery system*.ti,ab,kw. or ((industrial or automobile* or traffic or freeway or highway or roadway or motorway or diesel or vehicle*) adj3 (exhaust* or emission* or emit* or fume or fumes or vapo?r*)).ti,ab,kw.	264528
3	1 or 2	581968

4	(health* or disease* or outbreak* or infectio* or illness* or virus* or injur* or disorder* or epidemic* or pandemic* or fever* or symptoms).mp. or exp pregnancy complications/ or exp infant, Low Birth Weight/ or stillbirth/ or (deformit* or malformation* or cleft-palate* or cleft-lip* or birth-defect* or congenital heart defect* or spina-bifida or birth-outcome* or pregnancy-outcome* or still-birth or still-born* or stillborn or stillbirth* or low-birth-weight or (fetal-growth adj (restric* or retard*)) or preterm labo* or preterm birth or (hypertension and pregnan*) or preeclampsia).mp. or (((developmental* or intellectual* or learning or mental or cognitive or physical*) adj3 (disab* or impair* or delay* or disorder*)) or mental retard* or handicap* or special needs or down* syndrome or "fragile x" or autis* or asperger* or pervasive developmental disorder* or cerebral palsy or tourette* or tic-disorder* or attention-deficit* or adhd or epilep* or language-disorder* or neurodevelopment* or ((neuro or cognitive or physical*) adj5 development*)).mp. or stress, psychological/ or occupational stress/ or (stress-level* or high*-stress* or psychological-stress* or mental-stress* or burnout or psychiatric or depress* or anxiety or psychos* or stress-disorder* or PTSD or posttrauma* or trauma* or emotional-health).mp. or exp occupational diseases/ or (food-security or food-insecurity or nutrition or undernutrition or malnutrition or malnourish* or undernourish* or overnutrition or famine or food-availability or food-supply or ((nutrition* or vitamin) adj4 deficien*)).mp. or (underweight or thinness or overweight or obes*).mp. or exp diseases/ or exp food safety/ or dietary exposure/ or (food-safety or (food adj4 (contaminat* or poison*))).mp. or ((safe or clean or unsafe or contamina*) adj6 (drink* or potable) adj6 water).mp. or exp mortality/ or exp morbidity/ or pregnancy outcome/ or (mortalit* or death* or fatalit*).mp. or (respiratory or asthma* or allerg* or aeroallerg* or ((breathing or breathe) adj3 (issue* or problem* or difficult* or struggl* or trouble))).mp. or exp heat stress disorders/ or (heat-stress* or heat-exhaustion or heat-stroke* or heatstroke* or sun stroke* or sunstroke*).mp. or (cholera* or diarrhea* or diarrhoea* or dysentery or salmonella or pneumonia* or plasmodium* or babesi* or hantavirus* or giardia* or cryptosporidi* or leptospiros*).mp. or exp parasitic diseases/ or (Trichinos* or trichinellosis or Chikungunya or Dengue or Lymphatic-filariasis or Rift-Valley-fever or Yellow-fever or Zika or Malaria or japanese-encephalitis or tick-borne or West-Nile-fever or Leishmaniasis or Sandfly-fever or phelebotomus-fever or haemorrhagic-fever or Lyme-disease or Relapsing-fever or borreliosis or Rickettsial-diseases or spotted-fever or Q-fever or Tularaemia or Chagas or trypanosomiasis or Sleeping-sickness or Plague or Rickettsiosis or Onchocerciasis or river-blindness or Schistosomiasis or bilharziasis or Typhus or louse-borne-relapsing-fever).mp.	14775744

5	(canad* or "british columbia" or alberta* or saskatchewan or manitoba* or ontario or quebec or (new brunswick not new jersey) or nouveau brunswick or nova scotia or nouvelle ecosse or prince edward island or newfoundland or labrador or nunavut or nwt or northwest territories or yukon or nunavik or inuvialuit).mp,jw,nw. or (Abbotsford or Airdrie or Ajax or Aurora or Barrie or Belleville or Blainville or Brampton or Brantford or Brossard or Burlington or Burnaby or Caledon or Calgary or Cape Breton or Chatham Kent or Chilliwack or Clarington or Coquitlam or Drummondville or Edmonton or Fredericton or Fort McMurray or Gatineau or Granby or Grande Prairie or Sudbury or Guelph or Halton Hills or Iqaluit or Inuvik or Kamloops or Kawartha Lakes or Kelowna or Kingston or Kitchener or Langley or Laval or Lethbridge or Levis or Longueuil or Maple Ridge or Markham or Medicine Hat or Milton or Mirabel or Mississauga or Moncton or Montreal or Nanaimo or New Westminster or Newmarket or Niagara Falls or Norfolk County or North Bay or North Vancouver or North Vancouver or Oakville or Oshawa or Ottawa or Peterborough or Pickering or Port Coquitlam or Prince George or Quebec City or Red Deer or Regina or Repentigny or Richmond or Richmond Hill or Saanich or Saguenay or Saint John or Saint-Hyacinthe or Saint-Jean-sur-Richelieu or Saint-Jerome or Sarnia or Saskatoon or Sault Ste Marie or Sherbrooke or St Albert or St Catharines or St John's or Strathcona County or Surrey or Terrebonne or Thunder Bay or Toronto or Trois-Rivieres or Vancouver or Vaughan or ((Cambridge or (Halifax or Hamilton or London or Victoria or Waterloo or Welland or Whitby or Windsor)) not (UK or Britain or United Kingdom or England or Australia)) or Whitehorse or Winnipeg or Wood Buffalo or Yellowknife).ti,ab,kw.	505803
6	exp geriatrics/ or aged/ or aged hospital patient/ or exp elderly care/ or frail elderly/ or gerontology/ or institutionalized elderly/ or very elderly/ or ("aging in place" or elders or elderly or geriatric* or gerodontic* or old age or (seniors not "high school") or older adult* or centenarian* or nonagenarian* or octogenarian* or septuagenarian* or sexagenarian* or dottering or decrepit or tottering or overaged or "oldest old").mp.	3188044
7	exp Pregnancy Complications/ or exp Pregnancy/ or exp Pregnant Women/ or exp Labor, Obstetric/ or exp Delivery, Obstetric/ or exp Obstetric Labor, Premature/ or exp Obstetric Labor Complications/ or Prenatal Care/ or (obstetric* or prenatal* or pregnan* or gestat* or perinatal or antenatal or parturi* or childbirth or (labo?r adj3 delivery)).mp.	1194538
8	adolescent/ or exp child/ or child, preschool/ or infant/	3224561
9	child/ or child, abandoned/ or child, adopted/ or exp child, exceptional/ or "child of impaired parents"/ or child, foster/ or child, orphaned/ or child, unwanted/	1678057

10	(fetus* or fetal or foetus* or foetal* or "in utero" or pediatric* or paediatric* or child* or newborn* or congenital* or infan* or baby or babies or neonat* or pre term or preterm* or premature birth or NICU or preschool* or pre school* or kindergarten* or elementary school* or nursery school* or schoolchild* or toddler* or boy or boys or girl* or middle school* or pubescen* or juvenile* or teen* or youth* or high school* or adolesc* or prepubesc* or pre pubesc*).mp. or (child* or adolesc* or pediat* or paediat*).jn.	4766683
11	exp "Emigrants and Immigrants"/ or Refugees/ or (immigrant* or immigration or emigrant* or emigration or refugee* or asylum seeker* or asylee* or displaced person* or "incomer*" or "in comer*" or "new comer*" or newcomer* or migrant* or resettler*).mp.	82844
12	(exp Indians, North American/ or exp Inuits/ or exp Health Services, Indigenous/ or exp Ethnopharmacology/ or Athapaskan.mp. or Saulteaux.mp. or Wakashan.mp. or Cree.mp. or Dene.mp. or Inuit.mp. or Inuk.mp. or Inuvialuit*.mp. or Haida.mp. or Ktunaxa.mp. or Tsimshian.mp. or Gitsxan.mp. or Nisga'a.mp. or Haisla.mp. or Heiltsuk.mp. or Oweenkeno.mp. or Kwakwaka'wakw.mp. or Nuu chah nulth.mp. or Tsilhqot'in.mp. or Dakelh.mp. or Wet'suwet'en.mp. or Sekani.mp. or Dunne-za.mp. or Dene.mp. or Tahltan.mp. or Kaska.mp. or Tagish.mp. or Tutchone.mp. or Nuxalk.mp. or Salish.mp. or Stl'atlimc.mp. or Nlaka'pamux.mp. or Okanagan.mp. or Sec wepmc.mp. or Tlingit.mp. or Anishinaabe.mp. or Blackfoot.mp. or Nakoda.mp. or Tasttine.mp. or Tsuu T'inia.mp. or Gwich'in.mp. or Han.mp. or Tagish.mp. or Tutchone.mp. or Algonquin.mp. or Nipissing.mp. or Ojibwa.mp. or Potawatomi.mp. or Innu.mp. or Maliseet.mp. or Mi'kmaq.mp. or Micmac.mp. or Passamaquoddy.mp. or Haudenosaunee.mp. or Cayuga.mp. or Mohawk.mp. or Oneida.mp. or Onodaga.mp. or Seneca.mp. or Tuscarora.mp. or Wyandot.mp. or Aboriginal*.mp. or Indigenous*.mp. or Metis.mp. or red road.mp. or "on reserve".mp. or off-reserve.mp. or First Nation.mp. or First Nations.mp. or Amerindian.mp. or (urban adj3 (Indian* or Native* or Aboriginal*)).mp. or ethnomedicine.mp. or country food*.mp. or residential school*.mp. or ((exp Medicine, Traditional/ or traditional medicine*.mp.) not Chinese.mp.) or exp Shamanism/ or shaman*.mp. or traditional heal*.mp. or traditional food*.mp. or medicine man.mp. or medicine woman.mp. or autochtone*.mp. or (Native* adj1 (man or men or women or woman or boy* or girl* or adolescent* or youth or youths or person* or adult or people* or Indian* or Nation or tribe* or tribal or band or bands)).mp.) and (exp Canada/ or (Canad* or British Columbia or Columbie Britannique or Alberta or Saskatchewan or Manitoba or Ontario or Quebec or Nova Scotia or New Brunswick or Newfoundland or Labrador or Prince Edward Island or Yukon Territory or NWT or Northwest Territories or Nunavut or Nunavik or Nunatsiavut or NunatuKavut).mp.)	7400

13	occupational exposure/ or ((agricultur* or forest* or farm* or field or fields) adj3 (work* or labo?*)).mp. or (((((arborist* or archaeologist* or fruit) adj3 picker*) or farm labo?r or berry pick* or cowboy* or cowgirl* or "deck hand" or dog walker* or farmworker* or fisher* worker* or fisherm?n or firefighter* or fire fighter* or farmer* or farmhand* or forester or flagm?n or flagwom?n or greens keeper* or grounds keeper* or gardener* or garbagewom* or (garbage adj3 (man or men or women or woman or collector* or worker*)) or herder* or hunter* or letter carrier* or lifeguard* or landscaper* or mail carrier* or marine biologist* or mariner* or ((outdoor* or outside or "out of doors") adj3 (work* or employment or construction* or job or jobs)) or playground supervisor* or power line technician* or ski instructor* or ski patrol* or ranger or rangers or "rig hand*" or sailor* or parks worker* or shepherd* or smoke jumper* or stoop labo?r* or trapper* or tree planter* or vegetable) adj3 picker*) or wildlife biologist*).mp. [mp=title, abstract, original title, name of substance word, subject heading word, floating sub-heading word, keyword heading word, organism supplementary concept word, protocol supplementary concept word, rare disease supplementary concept word, unique identifier, synonyms]	83677
14	6 or 7 or 8 or 9 or 10 or 11 or 12 or 13 or Vulnerable Populations/	7909385
15	3 and 4 and 5 and 14	2274
16	Animals/ not (Animals/ and Humans/)	4667692
17	15 not 16	2178
18	(smoking or smoker* or "who smoke" or squirrel* or (newcastle adj2 ottawa)).mp.	312283
19	17 not 18	1464
20	remove duplicates from 19	1457

Embase <1974 to 2020 May 22>

#	Search Statement	Results

1	weather/ or Climate/ or climatic processes/ or ((exp climate change/ or cyclonic storms/ or droughts/ or floods/ or greenhouse effect/ or temperature/) and (ambient or season* or climate or climatic or weather or summer or winter).mp.) or extreme heat/ or hot temperature/ or (((climat* not (political-climate or organizational-climate or economic-climate or financial-climate)) or weather) adj3 (chang* or disrupt* or volati* or instabilit* or unstable or variable or variability or vulnerab*)).mp. or ((chang* or decline* or decrease* or increase*) adj3 humidity).mp. or ((global* or climate) adj2 warm*).mp. or ((climat* or weather) adj (conditions or factors or driven)).mp. or (greenhouse gas* or greenhouse effect* or carbon emission* or carbon dioxide emission* or CO2 emission*).mp. or (((extreme or severe) adj (weather or heat or temperature*)) or hot-weather or heat-wave* or heatwave or high temperatures or ((higher or warm* or hot*) adj3 temperature*)).mp. or ((annual* or daily or month* or ambient or environmental or season* or climate or climatic or weather or summer or winter) adj4 temperature*).mp. or (warm-season* or warm*-month* or (unseasonabl* adj3 (warm or hot or heat or humid* or cold or high or low))).mp. or (El Nino or la nina).mp. or (rain or rains or rainstorm* or raining or rainfall or ((high or heavy or extreme or severe or daily or levels) adj2 precipitation) or UV-index or flooding or floods or waterlogging or (drought* not drought-resistan*) or desertifcation or hurricane* or cyclone* or tornado* or superstorm* or dust storm or storm surge* or ice storm* or ((storm or storms) and disaster*) or monsoon*).mp. or (forest fire* or wildfire* or wild fire* or fire disaster* or (uncontrolled adj3 fire*)).mp. or ((sea level* adj3 (rise or rises or rising or increase or increasing or increases)) or ((polar or permafrost or ice-cap or glacier*) adj3 (melt* or retreat* or reced* or thaw*))).mp. or famine*.mp. [mp=title, abstract, heading word, drug trade name, original title, device manufacturer, drug manufacturer, device trade name, keyword, floating subheading word, candidate term word]	301167

2	exp Air Pollution/ or Traffic-Related Pollution/ or greenhouse gases/ or stratospheric ozone/ or ((gases/ or ammonia/ or carbon dioxide/ or carbon monoxide/ or chlorine/ or greenhouse gases/ or hydrogen sulfide/ or nitrogen/ or nitrogen oxides/ or nitrogen dioxide/ or nitrous oxide/ or ozone/ or sulfur dioxide/ or carbon monoxide*.mp. or hydrogen sulfide*.mp. or hydrogen sulphide*.mp. or H2S.mp. or nitrogen oxide*.mp. or volatile organic*.mp. or voc.mp. or nox.mp. or sulfur dioxide*.mp. or SO2.mp. or sulfur oxide*.mp. or sulphur dioxide*.mp. or sulphur oxide*.mp.) and (exp Pollution/ or pollut*.mp. or emit*.mp. or emission*.mp. or contaminat*.mp. or decontaminat*.mp.)) or ((air or atmospher*) adj3 (pollut* or quality or wast* or contamin* or emission* or immission* or effluent*or acidification or contaminat* or degrad* or decontaminat* or purif* or restor*)).mp. or ((gas or gases) adj3 (toxic* or noxious or releas* or purifi* or scrub* or emit* or waste* or vapo?r)).mp. or (acid* adj3 (deposition* or rain* or snow or fog or mist or precipitation or hail or sleet)).mp. or afterburning.mp. or biogas.mp. or (burning adj3 fossil fuel*).mp. or ((chimney or stack) adj3 height).mp. or dust.mp. or fallout.mp. or fall out.mp. or flaring.mp. or fly ash.mp. or electrosmog.mp. or (greenhouse adj3 (gas or effect or effects or gases or emission*)).mp. or incineration.mp. or odor.mp. or odour.mp. or olfactory pollution.mp. or particulate*.mp. or smog.mp. or smoke.mp. or soot.mp. or sick building syndrome.mp. or vapo?r recovery system*.mp. or ((industrial or automobile* or traffic or freeway or highway or roadway or motorway or diesel or vehicle*) adj3 (exhaust* or emission* or emit* or fume or fumes or vapo?r*)).mp. [mp=title, abstract, heading word, drug trade name, original title, device manufacturer, drug manufacturer, device trade name, keyword, floating subheading word, candidate term word]	460010
3	1 or 2	702521

4	(health* or disease* or outbreak* or infectio* or illness* or virus* or injur* or disorder* or epidemic* or pandemic* or fever* or symptoms).mp. or exp pregnancy complications/ or exp infant, Low Birth Weight/ or stillbirth/ or (deformit* or malformation* or cleft-palate* or cleft-lip* or birth-defect* or congenital heart defect* or spina-bifida or birth-outcome* or pregnancy-outcome* or still-birth or still-born* or stillborn or stillbirth* or low-birth-weight or (fetal-growth adj (restric* or retard*)) or preterm labo* or preterm birth or (hypertension and pregnan*) or preeclampsia).mp. or (((developmental* or intellectual* or learning or mental or cognitive or physical*) adj3 (disab* or impair* or delay* or disorder*)) or mental retard* or handicap* or special needs or down* syndrome or "fragile x" or autis* or asperger* or pervasive developmental disorder* or cerebral palsy or tourette* or tic-disorder* or attention-deficit* or adhd or epilep* or language-disorder* or neurodevelopment* or ((neuro or cognitive or physical*) adj5 development*)).mp. or stress, psychological/ or occupational stress/ or (stress-level* or high*-stress* or psychological-stress* or mental-stress* or burnout or psychiatric or depress* or anxiety or psychos* or stress-disorder* or PTSD or posttrauma* or trauma* or emotional-health).mp. or exp occupational diseases/ or (food-security or food-insecurity or nutrition or undernutrition or malnutrition or malnourish* or undernourish* or overnutrition or famine or food-availability or food-supply or ((nutrition* or vitamin) adj4 deficien*)).mp. or (underweight or thinness or overweight or obes*).mp. or exp diseases/ or exp food safety/ or dietary exposure/ or (food-safety or (food adj4 (contaminat* or poison*))).mp. or ((safe or clean or unsafe or contamina*) adj6 (drink* or potable) adj6 water).mp. or exp mortality/ or exp morbidity/ or pregnancy outcome/ or (mortalit* or death* or fatalit*).mp. or (respiratory or asthma* or allerg* or aeroallerg* or ((breathing or breathe) adj3 (issue* or problem* or difficult* or struggl* or trouble))).mp. or exp heat stress disorders/ or (heat-stress* or heat-exhaustion or heat-stroke* or heatstroke* or sun stroke* or sunstroke*).mp. or (cholera* or diarrhea* or diarrhoea* or dysentery or salmonella or pneumonia* or plasmodium* or babesi* or hantavirus* or giardia* or cryptosporidi* or leptospiros*).mp. or exp parasitic diseases/ or (Trichinos* or trichinellosis or Chikungunya or Dengue or Lymphatic-filariasis or Rift-Valley-fever or Yellow-fever or Zika or Malaria or japanese-encephalitis or tick-borne or West-Nile-fever or Leishmaniasis or Sandfly-fever or phelebotomus-fever or haemorrhagic-fever or Lyme-disease or Relapsing-fever or borreliosis or Rickettsial-diseases or spotted-fever or Q-fever or Tularaemia or Chagas or trypanosomiasis or Sleeping-sickness or Plague or Rickettsiosis or Onchocerciasis or river-blindness or Schistosomiasis or bilharziasis or Typhus or louse-borne-relapsing-fever).mp.	24335656

5	(exp Canada/ or ((canad* or "british columbia" or alberta* or saskatchewan or manitoba* or ontario or quebec or (new brunswick not new jersey) or nouveau brunswick or nova scotia or nouvelle ecosse or prince edward island or newfoundland or labrador or nunavut or nwt or northwest territories or yukon or nunavik or inuvialuit).mp. or (Abbotsford or Airdrie or Ajax or Aurora or Barrie or Belleville or Blainville or Brampton or Brantford or Brossard or Burlington or Burnaby or Caledon or Calgary or Cape Breton or Chatham Kent or Chilliwack or Clarington or Coquitlam or Drummondville or Edmonton or Fredericton or Fort McMurray or Gatineau or Granby or Grande Prairie or Sudbury or Guelph or Halton Hills or Iqaluit or Inuvik or Kamloops or Kawartha Lakes or Kelowna or Kingston or Kitchener or Langley or Laval or Lethbridge or Levis or Longueuil or Maple Ridge or Markham or Medicine Hat or Milton or Mirabel or Mississauga or Moncton or Montreal or Nanaimo or New Westminster or Newmarket or Niagara Falls or Norfolk County or North Bay or North Vancouver or North Vancouver or Oakville or Oshawa or Ottawa or Peterborough or Pickering or Port Coquitlam or Prince George or Quebec City or Red Deer or Regina or Repentigny or Richmond or Richmond Hill or Saanich or Saguenay or Saint John or Saint-Hyacinthe or Saint-Jean-sur-Richelieu or Saint-Jerome or Sarnia or Saskatoon or Sault Ste Marie or Sherbrooke or St Albert or St Catharines or St John's or Strathcona County or Surrey or Terrebonne or Thunder Bay or Toronto or Trois-Rivieres or Vancouver or Vaughan or (Cambridge or (Halifax or Hamilton or London or Victoria or Waterloo or Welland or Whitby or Windsor)) or Whitehorse or Winnipeg or Wood Buffalo or Yellowknife).ti,ab.)) not (UK or Britain or United Kingdom or Scotland or US or United States or USA or India or New Zealand or England or Australia).mp.	346197
6	exp geriatrics/ or aged/ or aged hospital patient/ or exp elderly care/ or frail elderly/ or gerontology/ or institutionalized elderly/ or very elderly/ or ("aging in place" or elders or elderly or geriatric* or gerodontic* or old age or (seniors not "high school") or older adult* or centenarian* or nonagenarian* or octogenarian* or septuagenarian* or sexagenarian* or dottering or decrepit or tottering or overaged or "oldest old").mp.	3121015
7	juvenile/ or exp adolescent/ or exp child/ or exp postnatal development/ or (pediatric* or paediatric* or child* or newborn* or congenital* or infan* or baby or babies or neonat* or pre term or preterm* or premature birth or NICU or preschool* or pre school* or kindergarten* or elementary school* or nursery school* or schoolchild* or toddler* or boy or boys or girl* or middle school* or pubescen* or juvenile* or teen* or youth* or high school* or adolesc* or prepubesc* or pre pubesc*).mp. or (child* or adolesc* or pediat* or paediat*).jn.	4657251
8	(fetus* or fetal or foetus* or foetal* or "in utero").mp.	505191

9	exp Refugees/ or (immigrant* or immigration or emigrant* or emigration or refugee* or asylum seeker* or asylee* or displaced person* or "incomer*" or "in comer*" or "new comer*" or newcomer* or migrant* or resettler*).mp.	79847
10	exp Indians, North American/ or exp Inuits/ or exp Health Services, Indigenous/ or exp Ethnopharmacology/ or Athapaskan.mp. or Saulteaux.mp. or Wakashan.mp. or Cree.mp. or Dene.mp. or Inuit.mp. or Inuk.mp. or Inuvialuit*.mp. or Haida.mp. or Ktunaxa.mp. or Tsimshian.mp. or Gitsxan.mp. or Nisga'a.mp. or Haisla.mp. or Heiltsuk.mp. or Oweenkeno.mp. or Kwakwaka'wakw.mp. or Nuu chah nulth.mp. or Tsilhqot'in.mp. or Dakelh.mp. or Wet'suwet'en.mp. or Sekani.mp. or Dunne-za.mp. or Dene.mp. or Tahltan.mp. or Kaska.mp. or Tagish.mp. or Tutchone.mp. or Nuxalk.mp. or Salish.mp. or Stl'atlimc.mp. or Nlaka'pamux.mp. or Okanagan.mp. or Sec wepmc.mp. or Tlingit.mp. or Anishinaabe.mp. or Blackfoot.mp. or Nakoda.mp. or Tasttine.mp. or Tsuu T'inia.mp. or Gwich'in.mp. or Han.mp. or Tagish.mp. or Tutchone.mp. or Algonquin.mp. or Nipissing.mp. or Ojibwa.mp. or Potawatomi.mp. or Innu.mp. or Maliseet.mp. or Mi'kmaq.mp. or Micmac.mp. or Passamaquoddy.mp. or Haudenosaunee.mp. or Cayuga.mp. or Mohawk.mp. or Oneida.mp. or Onodaga.mp. or Seneca.mp. or Tuscarora.mp. or Wyandot.mp. or Aboriginal*.mp. or Indigenous*.mp. or Metis.mp. or red road.mp. or "on reserve".mp. or off-reserve.mp. or First Nation.mp. or First Nations.mp. or Amerindian.mp. or (urban adj3 (Indian* or Native* or Aboriginal*)).mp. or ethnomedicine.mp. or country food*.mp. or residential school*.mp. or ((exp Medicine, Traditional/ or traditional medicine*.mp.) not Chinese.mp.) or exp Shamanism/ or shaman*.mp. or traditional heal*.mp. or traditional food*.mp. or medicine man.mp. or medicine woman.mp. or autochtone*.mp. or (Native* adj1 (man or men or women or woman or boy* or girl* or adolescent* or youth or youths or person* or adult or people* or Indian* or Nation or tribe* or tribal or band or bands)).mp.	156651
11	occupational exposure/ or ((agricultur* or forest* or farm* or field or fields) adj3 (work* or labo?*)).mp. or (((((arborist* or archaeologist* or fruit) adj3 picker*) or farm labo?r or berry pick* or cowboy* or cowgirl* or "deck hand" or dog walker* or farmworker* or fisher* worker* or fisherm?n or firefighter* or fire fighter* or farmer* or farmhand* or forester or flagm?n or flagwom?n or greens keeper* or grounds keeper* or gardener* or garbagewom* or (garbage adj3 (man or men or women or woman or collector* or worker*)) or herder* or hunter* or letter carrier* or lifeguard* or landscaper* or mail carrier* or marine biologist* or mariner* or ((outdoor* or outside or "out of doors") adj3 (work* or employment or construction* or job or jobs)) or playground supervisor* or power line technician* or ski instructor* or ski patrol* or ranger or rangers or "rig hand*" or sailor* or parks worker* or shepherd* or smoke jumper* or stoop labo?r* or trapper* or tree planter* or vegetable) adj3 picker*) or wildlife biologist*).mp. [mp=title, abstract, heading word, drug trade name, original title, device manufacturer, drug manufacturer, device trade name, keyword, floating subheading word, candidate term word]	125823

12	6 or 7 or 8 or 9 or 10 or 11 or At Risk Population/	7836083
13	3 and 4 and 5 and 12	2290
14	(smoker* or smoking or "who smoke" or squirrel*).mp.	498789
15	exp animal/	25497351
16	exp human/	20875431
17	15 and 16	20875431
18	15 not 17	4621920
19	14 or 18	5108004
20	13 not 19	1486
21	remove duplicates from 20	1468

Global Health <1910 to 2020 Week 20>

#	Search Statement	Results

1	(ambient or season* or climate or climatic or weather or summer or winter or (((climat* not (political-climate or organizational-climate or economic-climate or financial-climate)) or weather) adj3 (chang* or disrupt* or volati* or instabilit* or unstable or variable or variability or vulnerab*)) or ((chang* or decline* or decrease* or increase*) adj3 humidity) or ((global* or climate) adj2 warm*) or ((climat* or weather) adj (conditions or factors or driven)) or (greenhouse gas* or greenhouse effect* or carbon emission* or carbon dioxide emission* or CO2 emission*) or (((extreme or severe) adj (weather or heat or temperature*)) or hot-weather or heat-wave* or heatwave or high temperatures or ((higher or warm* or hot*) adj3 temperature*)) or ((annual* or daily or month* or ambient or environmental or season* or climate or climatic or weather or summer or winter) adj4 temperature*) or (warm-season* or warm*-month* or (unseasonabl* adj3 (warm or hot or heat or humid* or cold or high or low))) or (El Nino or la nina) or (rain or rains or rainstorm* or raining or rainfall or ((high or heavy or extreme or severe or daily or levels) adj2 precipitation) or UV-index or flooding or floods or waterlogging or (drought* not drought-resistan*) or desertifcation or hurricane* or cyclone* or tornado* or superstorm* or dust storm or storm surge* or ice storm* or ((storm or storms) and disaster*) or monsoon*) or (forest fire* or wildfire* or wild fire* or fire disaster* or (uncontrolled adj3 fire*)) or ((sea level* adj3 (rise or rises or rising or increase or increasing or increases)) or ((polar or permafrost or ice-cap or glacier*) adj3 (melt* or retreat* or reced* or thaw*))) or famine*).mp.	194810
2	(Arctic amplification or Arctic shrinkage or carbon footprint or carbon offset or carbon sequestration or carbon sink or carbon sinks or (chlorofluorocarbon* adj2 (release or concentration* or atmosphere*)) or (climate adj2 (change* or model?ing or predict* or resilience or sensitivity)) or deglaciation or desertification or disaster risk* or drought or (earth adj2 warming) or El Nino or La Nina or emissions reduc* or emissions trading or ((glacial or glacier) adj3 (retreat* or melt*)) or global radiation or global temperature* or global warming or greenhouse effect or greenhouse gas or heat wave* or ice cap melt* or ice mass loss* or Kyoto Protocol or (ozone adj2 hole) or Paris Accord or permafrost melt* or polar amplification or polar ice melt* or sea ice shrinkage or sea level rise* or sea surface warming or thermohaline circulation or ((extreme or severe) adj2 (temperature* or weather* or storm*)) or wildfire*).mp. [mp=abstract, title, original title, broad terms, heading words, identifiers, cabicodes]	19251

3	1 or 2	195701
4	((canad* or "british columbia" or alberta* or saskatchewan or manitoba* or ontario or quebec or (new brunswick not new jersey) or nouveau brunswick or nova scotia or nouvelle ecosse or prince edward island or newfoundland or labrador or nunavut or nwt or northwest territories or yukon or nunavik or inuvialuit).mp. or (Abbotsford or Airdrie or Ajax or Aurora or Barrie or Belleville or Blainville or Brampton or Brantford or Brossard or Burlington or Burnaby or Caledon or Calgary or Cape Breton or Chatham Kent or Chilliwack or Clarington or Coquitlam or Drummondville or Edmonton or Fredericton or Fort McMurray or Gatineau or Granby or Grande Prairie or Sudbury or Guelph or Halton Hills or Iqaluit or Inuvik or Kamloops or Kawartha Lakes or Kelowna or Kingston or Kitchener or Langley or Laval or Lethbridge or Levis or Longueuil or Maple Ridge or Markham or Medicine Hat or Milton or Mirabel or Mississauga or Moncton or Montreal or Nanaimo or New Westminster or Newmarket or Niagara Falls or Norfolk County or North Bay or North Vancouver or North Vancouver or Oakville or Oshawa or Ottawa or Peterborough or Pickering or Port Coquitlam or Prince George or Quebec City or Red Deer or Regina or Repentigny or Richmond or Richmond Hill or Saanich or Saguenay or Saint John or Saint-Hyacinthe or Saint-Jean-sur-Richelieu or Saint-Jerome or Sarnia or Saskatoon or Sault Ste Marie or Sherbrooke or St Albert or St Catharines or St John's or Strathcona County or Surrey or Terrebonne or Thunder Bay or Toronto or Trois-Rivieres or Vancouver or Vaughan or (Cambridge or (Halifax or Hamilton or London or Victoria or Waterloo or Welland or Whitby or Windsor)) or Whitehorse or Winnipeg or Wood Buffalo or Yellowknife).ti,ab.) not (UK or Britain or United Kingdom or Scotland or US or United States or USA or India or New Zealand or England or Australia).mp. [mp=abstract, title, original title, broad terms, heading words, identifiers, cabicodes]	54465
5	(elders or elderly or geriatric* or old age or oldest age* or (seniors not "high school") or old* adult* or oldest patient* or "old old" or "very old" or centenarian* ornonagenarian* or octogenarian* or septuagenarian* or sexagenarian* or dottering or decrepit or tottering or late life or overaged or "oldest old").mp.	77193

6	(pediatric* or paediatric* or child* or newborn* or congenital* or infan* or baby or babies or neonat* or pre term or preterm* or premature birth or NICU or preschool* or pre school* or kindergarten* or elementary school* or nursery school* or schoolchild* or toddler* or boy or boys or girl* or middle school* or pubescen* or juvenile* or teen* or youth* or high school* or adolesc* or prepubesc* or pre pubesc*).mp. or (child* or adolesc* or pediat* or paediat*).jn.	615057
7	(fetus* or fetal or foetus* or foetal* or "in utero").mp.	45212
8	(immigrant* or immigration or emigrant* or emigration or refugee* or asylum seeker* or asylee* or displaced person* or "incomer*" or "in comer*" or "new comer*" or newcomer* or migrant* or resettler*).mp.	26410
9	(Athapaskan or Saulteaux or Wakashan or Cree or Dene or Inuit or Inuk or Inuvialuit* or Haida or Ktunaxa or Tsimshian or Gitsxan or Nisga'a or Haisla or Heiltsuk or Oweenkeno or Kwakwaka'wakw or Nuu chah nulth or Tsilhqot'in or Dakelh or Wet'suwet'en or Sekani or Dunne-za or Dene or Tahltan or Kaska or Tagish or Tutchone or Nuxalk or Salish or Stl'atlimc or Nlaka'pamux or Okanagan or Sec wepmc or Tlingit or Anishinaabe or Blackfoot or Nakoda or Tasttine or Tsuu T'inia or Gwich'in or Han or Tagish or Tutchone or Algonquin or Nipissing or Ojibwa or Potawatomi or Innu or Maliseet or Mi'kmaq or Micmac or Passamaquoddy or Haudenosaunee or Cayuga or Mohawk or Oneida or Onodaga or Seneca or Tuscarora or Wyandot or Aboriginal* or Indigenous* or Metis or red road or "on reserve" or off-reserve or First Nation or First Nations or Amerindian or (urban adj3 (Indian* or Native* or Aboriginal*)) or ethnomedicine or country food* or residential school*).mp. or ((exp Medicine, Traditional/ or traditional medicine*.mp.) not Chinese.mp.) or exp Shamanism/ or shaman*.mp. or traditional heal*.mp. or traditional food*.mp. or medicine man.mp. or medicine woman.mp. or autochtone*.mp. or (Native* adj1 (man or men or women or woman or boy* or girl* or adolescent* or youth or youths or person* or adult or people* or Indian* or Nation or tribe* or tribal or band or bands)).mp.	111861

10	(((agricultur* or forest* or farm* or field or fields) adj3 (work* or labo?*)) or (arborist* or archaeologist* or (fruit adj3 picker*) or farm labo?r or berry pick* or cowboy* or cowgirl* or "deck hand" or dog walker* or farmworker* or fisher* worker* or fisherm?n or firefighter* or fire fighter* or farmer* or farmhand* or forester or flagm?n or flagwom?n or greens keeper* or grounds keeper* or gardener* or garbagewom* or (garbage adj3 (man or men or women or woman or collector* or worker*)) or herder* or hunter* or letter carrier* or lifeguard* or landscaper* or mail carrier* or marine biologist* or mariner* or ((outdoor* or outside or "out of doors") adj3 (work* or employment or construction* or job or jobs)) or playground supervisor* or power line technician* or ski instructor* or ski patrol* or ranger or rangers or "rig hand*" or sailor* or parks worker* or shepherd* or smoke jumper* or stoop labo?r* or trapper* or tree planter* or (vegetable adj3 picker*) or wildlife biologist*)).mp. [mp=abstract, title, original title, broad terms, heading words, identifiers, cabicodes]	48956
11	5 or 6 or 7 or 8 or 9 or 10	856160
12	3 and 4 and 11	1327
13	(smoker* or smoking or "who smoke" or dog or dogs or cat or cats or squirrel* or ungulate* or deer or boar or ((Newcastle adj2 Ottawa) or "mating season" or "C/victoria" or "B/victoria" or (yamagata adj5 victoria))).mp.	178386
14	12 not 13	1145
15	remove duplicates from 14	1140

CINAHL Plus with Full Text

#	Query<br>NOTE: Expanders - Apply equivalent subjects<br>Search modes - Find all my search term	Results
S1	(health* or disease* or outbreak* or infectio* or illness* or virus* or injur* or disorder* or epidemic* or pandemic* or fever* or symptoms or (deformit* or malformation* or cleft-palate* or cleft-lip* or birth-defect* or congenital heart defect* or spina-bifida or birth-outcome* or pregnancy-outcome* or still-birth or still-born* or stillborn or stillbirth* or low-birth-weight or (fetal-growth Near (restric* or retard*)) or preterm labo* or preterm birth or (hypertension and pregnan*) or preeclampsia) or (((developmental* or intellectual* or learning or mental or cognitive or physical*) Near/3 (disab* or impair* or delay* or disorder*)) or mental retard* or handicap* or special needs or down* syndrome or "fragile x" or autis* or asperger* or pervasive developmental disorder* or cerebral palsy or tourette* or tic-disorder* or attention-deficit* or adhd or epilep* or language-disorder* or neurodevelopment* or ((neuro or cognitive or physical*) Near/5 development*)) Near/ 6 water) or (mortalit* or death* or fatalit*) or (respiratory or asthma* or allerg* or aeroallerg* or ((breathing or breathe) Near/3 (issue* or problem* or difficult* or struggl* or trouble))) or (heat-stress* or heat-exhaustion or heat-stroke* or heatstroke* or sun stroke* or sunstroke*) or (cholera* or diarrhea* or diarrhoea* or dysentery or salmonella or pneumonia* or plasmodium* or babesi* or hantavirus* or giardia* or cryptosporidi* or leptospiros*) or (Trichinos* or trichinellosis or Chikungunya or Dengue or Lymphatic-filariasis or Rift-Valley-fever or Yellow-fever or Zika or Malaria or japanese-encephalitis or tick-borne or West-Nile-fever or Leishmaniasis or Sandfly-fever or phelebotomus-fever or haemorrhagic-fever or Lyme-disease or Relapsing-fever or borreliosis or Rickettsial-diseases or spotted-fever or Q-fever or Tularaemia or Chagas or trypanosomiasis or Sleeping-sickness or Plague or Rickettsiosis or Onchocerciasis or river-blindness or Schistosomiasis or bilharziasis or Typhus or louse-borne-relapsing-fever)	4,065,961

S2	ti ((ambient OR season* OR seasonal* OR climate OR climatic OR weather OR summer OR winter OR (((climat* AND NOT (political-climate OR "organizational climate*" OR economic-climate OR financial-climate)) OR weather) N/3 (chang* OR disrupt* OR volati* OR instabilit* OR unstable OR variable OR variability OR vulnerab*)) OR ((chang* OR decline* OR decrease* OR increase*) N/3 humidity) OR ((global* OR climate) N/2 warm*) OR ((climat* OR weather) N/1 (conditions OR factors OR driven)) OR ("greenhouse gas*" OR "greenhouse effect*" OR "carbon emission*" OR "carbon dioxide emission*" OR "CO2 emission*"))) OR (((extreme OR severe) N/1 (weather OR heat OR hot* OR temperature*)) OR ((annual* OR daily OR month* OR ambient OR environmental OR season* OR climate OR climatic OR weather OR summer OR winter) N/4 temperature*) OR (warm-season* OR warm*-month* OR ((unseasonabl* OR unusual*) N/3 (warm OR hot OR heat OR humid* OR cold OR high OR low)))) OR ((("El Nino" OR "La Nina") OR (rain OR rains OR rainstorm* OR raining OR rainfall OR ((high OR heavy OR extreme OR severe OR daily OR levels) N/2 precipitation) OR uv-index OR flooding OR floods OR waterlogging OR (drought* AND NOT drought-resistan*) OR desertifcation OR hurricane* OR cyclone* OR tornado* OR superstorm* OR "dust storm*" OR "storm surge*" OR "ice storm*" OR ((storm OR storms) AND disaster*) OR monsoon*) OR ("forest fire*" OR wildfire* OR "wild fire*" OR "fire disaster*" OR (uncontrolled N/2 fire*)) OR (("sea level*" N/3 (rise OR rises OR rising OR increase OR increasing OR increases)) OR ((polar OR permafrost OR ice-cap OR glacier*) N/3 (melt* OR retreat* OR reced* OR thaw*))) OR famine*))	30,271
S3	ti (((carbon monoxide* or hydrogen sulfide* or hydrogen sulphide* or H2S or nitrogen oxide* or volatile organic* or voc or nox or sulfur dioxide* or sulfur oxide* or sulphur dioxide* or sulphur oxide*) and (pollut* or emit* or emission* or contaminat* or decontaminat*)) or ((air or atmospher*) N/3 (pollut* or quality or wast* or contamin* or emission* or immission* or effluent*or acidification or contaminat* or degrad* or decontaminat* or purif* or restor*)) or ((gas or gases) N/3 (toxic* or noxious or releas* or purifi* or scrub* or emit* or waste* or vapo?r)) or (acid* N/3 (deposition* or rain* or snow or fog or mist or precipitation or hail or sleet)) or afterburning or biogas or (burning adj3 fossil fuel*) or ((chimney or stack) N/3 height) or dust or fallout or fall out or flaring or fly ash or electrosmog or (greenhouse N/3 (gas or effect or effects or gases or emission*)) or incineration or odor or odour or olfactory pollution or particulate* or smog or smoke or soot or sick building syndrome or vapo?r recovery system* or ((industrial or automobile* or traffic or freeway or highway or roadway or motorway or diesel or vehicle*) N/3 (exhaust* or emission* or emit* or fume or fumes or vapo?r*)))	10,294

S4	(pediatric* or paediatric* or child* or newborn* or congenital* or infan* or baby or babies or neonat* or “pre-term” or preterm or “premature birth*” or NICU or preschool* or “pre-school*” or kindergarten* or “elementary school*” or “nursery school*” or schoolchild* or toddler* or boy or boys or girl* or “middle school*” or pubescen* or juvenile* or teen* or youth* or “high school*” or adolesc*or prepubesc* or “pre-pubesc*” or "(MH "Child+") OR (MH "Adolescence+") OR (MH "Minors (Legal)") or "(MH "Child Abuse, Sexual") OR (MH "Child Behavior Disorders+") OR (MH "Child, Medically Fragile") OR (MH "Child Day Care") OR (MH "Child Behavior+") OR (MH "Child Mortality") OR (MH "Child Passenger Safety") OR (MH "Child Development Disorders, Pervasive+") OR (MH "Child Custody") OR (MH "Child Abuse+") OR (MH "Child Nutritional Physiology+") OR (MH "Child Behavior Checklist")) OR SO (child* or pediatric* or paediatric* or adolescent)	1,209,406
S5	Saulteaux or Wakashan or Cree or Dene or Inuit or Inuk or Inuvialuit* or Haida or Ktunaxa or Tsimshian or Gitsxan or Nisga'a or Haisla or Heiltsuk or Oweenkeno or Kwakwaka'wakw or Nuu chah nulth or Tsilhqot'i. or Dakelh or Wet'suwet'en or Sekani or Dunne-za or Dene or Tahltan or Kaska or Tagish or Tutchone or Nuxalk or Salish or Stl'atlimc or Nlaka'pamux or Okanagan or Sec wepmc or Tlingit or Anishinaabe or Blackfoot or Nakoda or Tasttine or Tsuu T'inia or Gwich'in or Han or Tagish or Tutchone or Algonquin or Nipissing or Ojibwa or Potawatomi or Innu or Maliseet or Mi'kmaq or Micmac or Passamaquoddy or Haudenosaunee or Cayuga or Mohawk or Oneida or Onodaga or Seneca or Tuscarora or Wyandot or Aboriginal* or Indigenous* or Metis or red road or "on reserve" or off-reserve or First Nation or First Nations or Amerindian or "urban indian*" or "urban native*" or "country food*" or "residential school*" or shaman* or "traditional heal*" or "traditional food*" or "medicine man" or "medicine woman" or autochtone*	41,828

S6	arborist* or archaeologist* or "fruit picker*" or "farm labor*" or "farm labour*" or berry pick* or cowboy* or cowgirl* or "deck hand" or dog walker* or farmworker* or fisher* worker* or fisherm?n or firefighter* or fire fighter* or farmer* or farmhand* or forester or flagm* or flagwom* or "greens keeper*" or "grounds keeper*" or gardener* or garbagewom* or "garbage man"or "garbage men" or "garbage women"or "garbage woman" or garbage collector* or garbage worker* or herder* or hunter* or "letter carrier*" or lifeguard* or landscaper* or "mail carrier*" or "marine biologist*" or mariner* or "outdoor* work*" or "outdoor employment*" or "outdoor construction" or "outdoor job* or "outside work*" or "outside employment*" or "outside construction*" or "outside job*" or "out of doors work*" or "out of doors employment" or "out of doors job*" or "playground supervisor*"	19,691
S7	"power line technician*" or "ski instructor*" or "ski patrol*" or ranger or rangers or "rig hand*" or sailor* or "parks worker*" or shepherd* or "smoke jumper*" or "stoop labour*" OR "stoop labour*" or trapper* or "tree planter*" or "vegetable picker*" or "wildlife biologist*"	1,118
S8	S4 or S5 OR S6 OR S7	1,260,582
S9	AB (((carbon monoxide* or hydrogen sulfide* or hydrogen sulphide* or H2S or nitrogen oxide* or volatile organic* or voc or nox or sulfur dioxide* or sulfur oxide* or sulphur dioxide* or sulphur oxide*) and (pollut* or emit* or emission* or contaminat* or decontaminat*)) or ((air or atmospher*) N/3 (pollut* or quality or wast* or contamin* or emission* or immission* or effluent*or acidification or contaminat* or degrad* or decontaminat* or purif* or restor*)) or ((gas or gases) N/3 (toxic* or noxious or releas* or purifi* or scrub* or emit* or waste* or vapo?r)) or (acid* N/3 (deposition* or rain* or snow or fog or mist or precipitation or hail or sleet)) or afterburning or biogas or (burning adj3 fossil fuel*) or ((chimney or stack) N/3 height) or dust or fallout or fall out or flaring or fly ash or electrosmog or (greenhouse N/3 (gas or effect or effects or gases or emission*)) or incineration or odor or odour or olfactory pollution or particulate* or smog or smoke or soot or sick building syndrome or vapo?r recovery system* or ((industrial or automobile* or traffic or freeway or highway or roadway or motorway or diesel or vehicle*) N/3 (exhaust* or emission* or emit* or fume or fumes or vapo?r*)))	25,797

S10	AB ((ambient OR season* OR seasonal* OR climate OR climatic OR weather OR summer OR winter OR (((climat* AND NOT (political-climate OR "organizational climate*" OR economic-climate OR financial-climate)) OR weather) N/3 (chang* OR disrupt* OR volati* OR instabilit* OR unstable OR variable OR variability OR vulnerab*)) OR ((chang* OR decline* OR decrease* OR increase*) N/3 humidity) OR ((global* OR climate) N/2 warm*) OR ((climat* OR weather) N/1 (conditions OR factors OR driven)) OR ("greenhouse gas*" OR "greenhouse effect*" OR "carbon emission*" OR "carbon dioxide emission*" OR "CO2 emission*"))) OR (((extreme OR severe) N/1 (weather OR heat OR hot* OR temperature*)) OR ((annual* OR daily OR month* OR ambient OR environmental OR season* OR climate OR climatic OR weather OR summer OR winter) N/4 temperature*) OR (warm-season* OR warm*-month* OR ((unseasonabl* OR unusual*) N/3 (warm OR hot OR heat OR humid* OR cold OR high OR low)))) OR ((("El Nino" OR "La Nina") OR (rain OR rains OR rainstorm* OR raining OR rainfall OR ((high OR heavy OR extreme OR severe OR daily OR levels) N/2 precipitation) OR uv-index OR flooding OR floods OR waterlogging OR (drought* AND NOT drought-resistan*) OR desertifcation OR hurricane* OR cyclone* OR tornado* OR superstorm* OR "dust storm*" OR "storm surge*" OR "ice storm*" OR ((storm OR storms) AND disaster*) OR monsoon*) OR ("forest fire*" OR wildfire* OR "wild fire*" OR "fire disaster*" OR (uncontrolled N/2 fire*)) OR (("sea level*" N/3 (rise OR rises OR rising OR increase OR increasing OR increases)) OR ((polar OR permafrost OR ice-cap OR glacier*) N/3 (melt* OR retreat* OR reced* OR thaw*))) OR famine*))	48,709
S11	S2 OR S3 OR S9 OR S10	99,213
S12	TI ((((Cambridge or (Halifax or Hamilton or London or Victoria or Waterloo or Welland or Whitby or Windsor)) not (UK or Britain or United Kingdom or England or Australia)))) OR AB ((((Cambridge or (Halifax or Hamilton or London or Victoria or Waterloo or Welland or Whitby or Windsor)) not (UK or Britain or United Kingdom or England or Australia))))	21,630

S13	TI (canad* or "british columbia" or alberta* or saskatchewan or manitoba* or ontario or quebec or (new brunswick not new jersey) or nouveau brunswick or nova scotia or nouvelle ecosse or prince edward island or newfoundland or labrador or nunavut or nwt or northwest territories or yukon or nunavik or inuvialuit or Abbotsford or Airdrie or Ajax or Aurora or Barrie or Belleville or Blainville or Brampton or Brantford or Brossard or Burlington or Burnaby or Caledon or Calgary or Cape Breton or Chatham Kent or Chilliwack or Clarington or Coquitlam or Drummondville or Edmonton or Fredericton or Fort McMurray or Gatineau or Granby or Grande Prairie or Sudbury or Guelph or Halton Hills or Iqaluit or Inuvik or Kamloops or Kawartha Lakes or Kelowna or Kingston or Kitchener or Langley or Laval or Lethbridge or Levis or Longueuil or Maple Ridge or Markham or Medicine Hat or Milton or Mirabel or Mississauga or Moncton or Montreal or Nanaimo or New Westminster or Newmarket or Niagara Falls or Norfolk County or North Bay or North Vancouver or North Vancouver or Oakville or Oshawa or Ottawa or Peterborough or Pickering or Port Coquitlam or Prince George or Quebec City or Red Deer or Regina or Repentigny or Richmond or Richmond Hill or Saanich or Saguenay or Saint John or Saint-Hyacinthe or Saint-Jean-sur-Richelieu or Saint-Jerome or Sarnia or Saskatoon or Sault Ste Marie or Sherbrooke or St Albert or St Catharines or St John's or Strathcona County or Surrey or Terrebonne or Thunder Bay or Toronto or Trois-Rivieres or Vancouver or Vaughan or Whitehorse or Winnipeg or Wood Buffalo or Yellowknife) OR AB (canad* or "british columbia" or alberta* or saskatchewan or manitoba* or ontario or quebec or (new brunswick not new jersey) or nouveau brunswick or nova scotia or nouvelle ecosse or prince edward island or newfoundland or labrador or nunavut or nwt or northwest territories or yukon or nunavik or inuvialuit or Abbotsford or Airdrie or Ajax or Aurora or Barrie or Belleville or Blainville or Brampton or Brantford or Brossard or Burlington or Burnaby or Caledon or Calgary or Cape Breton or Chatham Kent or Chilliwack or Clarington or Coquitlam or Drummondville or Edmonton or Fredericton or Fort McMurray or Gatineau or Granby or Grande Prairie or Sudbury or Guelph or Halton Hills or Iqaluit or Inuvik or Kamloops or Kawartha Lakes or Kelowna or Kingston or Kitchener or Langley or Laval or Lethbridge or Levis or Longueuil or Maple Ridge or Markham or Medicine Hat or Milton or Mirabel or Mississauga or Moncton or Montreal or Nanaimo or New Westminster or Newmarket or Niagara Falls or Norfolk County or North Bay or North Vancouver or North Vancouver or Oakville or Oshawa or Ottawa or Peterborough or Pickering or Port Coquitlam or Prince George or Quebec City or Red Deer or Regina or Repentigny or Richmond or Richmond Hill or Saanich or Saguenay or Saint John or Saint-Hyacinthe or Saint-Jean-sur-Richelieu or Saint-Jerome or Sarnia or Saskatoon or Sault Ste Marie or Sherbrooke or St Albert or St Catharines or St John's or Strathcona County or Surrey or Terrebonne or Thunder Bay or Toronto or Trois-Rivieres or Vancouver or Vaughan or Whitehorse or Winnipeg or Wood Buffalo or Yellowknife)	98,264

S14	(MH "Canada+")	109,490
S15	S12 OR S13 OR S14	172,064
S16	S1 AND S9 AND S12 AND S15	963

SCOPUS Results =678

(((((TITLE-ABS-KEY (afterburning OR smog OR electrosmog OR flaring OR ordor OR odour OR "fly ash" OR "fall out" OR "acid rain" OR "acid precipitation" OR "airborne particulate")) OR (TITLE-ABS-KEY ((air OR atmosphere OR gas*) W/3 (emission* OR immission* OR quality OR pollut* OR vapor* OR vapour* OR purifi* OR fume*))) OR (TITLE-ABS-KEY (dust OR ((industrial OR traffic OR automobile OR diesel AND of AND vehicle OR roadway OR motorway OR highway) AND (emit* OR emiss* OR vapor* OR vapour* OR fume*)))) OR (TITLE-ABS-KEY ("workplace air" OR "factory air" OR "airborne pesticide*" OR "airborne herbicide*" OR "airborne insecticide*" OR "insecticide mist*" OR "pesticide mist*" OR "herbicide mist*")) OR (TITLE-ABS-KEY ("climat* change" OR global-climate* OR "climate event" OR "disaster storm*" OR "global warming" OR "evironmental change" OR "greenhouse gas*" OR "greenhouse effect*" OR "carbon emission*" OR "carbon dioxide emission*" OR "CO2 emission*" OR desertifcation OR hurricane* OR cyclone* OR tornado* OR superstorm* OR "dust storm*" OR "storm surge*" OR "ice storm*" OR monsoon* OR "extreme heat" OR "extreme temperature*" OR "extreme cold" OR wildfire* OR "fire disaster*" OR "uncontrolled fire*" OR "sea level rise" OR "permafrost melt" OR "sea level ris*" OR "glacial retreat" OR "ice cap melt*" OR "thermohaline conveyor" OR "extreme precipitation" OR waterlogging OR flood* OR "El Nino" OR "La Nina" OR uv-index OR uv-rays OR "ozone hole"))))) AND (((TITLE-ABS-KEY ((elders OR elderly OR geriatric* OR "old age" OR "senior citizens" OR "older adult*" OR centenarian* OR nonagenarian* OR octogenarian* OR septuagenarian* OR sexagenarian* OR dottering OR decrepit OR tottering OR overaged OR "oldest old" OR pediatric* OR paediatric* OR child* OR newborn* OR congenital* OR infan* OR baby OR babies OR neonat* OR pre-term OR preterm* OR "premature birth*" OR nicu OR preschool* OR pre-school* OR kindergarten* OR kindergarden* OR "elementary school*" OR "nursery school*" OR "day care*" OR schoolchild* OR toddler* OR boy OR boys OR girl* OR "middle school*" OR pubescen* OR juvenile* OR teen* OR youth* OR "high school*" OR adolesc* OR pre-pubesc* OR prepubesc* OR immigrant* OR immigration OR emmigrant* OR emmigration OR refugee* OR "asylum seeker*" OR asylee* OR "displaced person*" OR "incomer*" OR "in comer*" OR "new comer*" OR newcomer* OR childbearing OR childbirth OR pregnan* OR fertile OR fecund OR womanish OR womanly OR maternal OR vulnerable)))) OR (fetus* OR fetal OR foetus* OR foetal* OR "in utero") OR (TITLE-ABS-KEY (athapaskan OR saulteaux OR wakashan OR cree OR dene OR inuit OR inuk OR inuvialuit* OR haida OR ktunaxa OR tsimshian OR gitsxan OR "Nisga'a" OR haisla OR heiltsuk OR oweenkeno OR "Kwakwaka'wakw" OR "Nuu chah nulth" OR "Tsilhqot'in" OR dakelh

OR "Wet'suwet'en" OR sekani OR dunne-za OR dene OR tahltan OR kaska OR tagish OR tutchone OR nuxalk OR salish OR "Stl'atlimc" OR "Nlaka'pamux" OR okanagan OR "Sec wepmc" OR tlingit OR anishinaabe OR blackfoot OR nakoda OR tasttine OR "Tsuu T'inia" OR "Gwich'in" OR han OR tagish OR tutchone OR algonquin OR nipissing OR ojibwa OR potawatomi OR innu OR maliseet OR mi'kmaq OR micmac OR passamaquoddy OR haudenosaunee OR cayuga OR mohawk OR oneida OR onodaga OR seneca OR tuscarora OR wyandot OR aboriginal* OR indigenous* OR metis OR red AND road OR "on reserve" OR off-reserve OR "First Nation" OR "First Nations" OR amerindian OR (urban W/3 (indian* OR native* OR aboriginal*)) OR ethnomedicine OR "country food*" OR "residential school*") OR ((traditional AND medicine*) not AND chinese) OR shaman* OR "traditional heal*" OR "traditional food*" OR "medicine man" OR "medicine woman" OR autochtone* OR (native* W/1 (man OR men OR women OR woman OR boy* OR girl* OR adolescent* OR youth OR youths OR person* OR adult OR people* OR indian* OR nation OR tribe* OR tribal OR band OR bands))) OR (TITLE-ABS-KEY (pregnan* OR "new mother*" OR puerperium OR obstetric* OR "labor and delivery" OR "labour and delivery" OR partus OR gravidida OR "peri natal*" OR perinatal OR postpartum OR "post-partum" OR antinatal* OR "anti natal")) OR (TITLE-ABS-KEY (((agricultur* OR forest* OR farm* OR field OR fields) W/3 (work* OR labo?*)) OR (arborist* OR archaeologist* OR (fruit W/3 picker*) OR "farm labo?r" OR "berry pick*" OR cowboy* OR cowgirl* OR "deck hand" OR "dog walker*" OR farmworker* OR "fisher* worker*" OR fisherm?n OR firefighter* OR "fire fighter*" OR farmer* OR farmhand* OR forester OR flagm?n OR flagwom?n OR "greens keeper*" OR "grounds keeper*" OR "grounds W/3 worker*" OR gardener* OR garbagewom* OR (garbage W/3 (man OR men OR women OR woman OR collector* OR worker*)) OR herder* OR hunter* OR "letter carrier*" OR lifeguard* OR landscaper* OR "mail carrier*" OR "marine biologist*" OR mariner* OR ((outdoor* OR outside OR "out of doors") W/3 (work* OR employment OR construction* OR job OR jobs)) OR "playground supervisor*" OR "power line technician*" OR "ski instructor*" OR "ski patrol*" OR ranger OR rangers OR "rig hand*" OR sailor* OR "parks worker*" OR shepherd* OR "smoke jumper*" OR "stoop labo?r*" OR trapper* OR "tree planter*" OR (vegetable W/3 picker*) OR wildlife AND biologist*)))) AND ((TITLE-ABS-KEY ((canad* OR "british columbia" OR alberta* OR saskatchewan OR manitoba* OR ontario OR quebec OR "new brunswick" OR "nouveau brunswick" OR "nova scotia" OR "nouvelle ecosse" OR "prince edward island" OR newfoundland OR labrador OR nunavut OR nwt OR "northwest territories" OR yukon OR nunavik OR inuvialuit)))) AND (((TITLE-ABS-KEY (health* OR disease* OR outbreak* OR infectio* OR illness* OR virus* OR injur* OR disorder* OR epidemic* OR pandemic* OR fever* OR symptoms OR (deformit* OR malformation* OR cleft-palate* OR cleft-lip* OR birth-defect* OR congenital AND heart AND defect* OR spina-bifida OR birth-outcome* OR pregnancy-outcome* OR still-birth OR still-born* OR stillborn OR stillbirth* OR low-birth-weight OR (fetal-growth W/3 (restric* OR retard*)) OR preterm AND labo* OR preterm AND birth OR (hypertension AND pregnan*) OR preeclampsia) OR (((developmental* OR intellectual* OR learning OR mental OR cognitive OR physical*) W/3 (disab* OR impair* OR delay* OR disorder*)) OR mental AND retard* OR handicap* OR special AND needs OR down* AND syndrome OR "fragile x" OR autis* OR asperger* OR pervasive AND developmental AND disorder* OR cerebral AND palsy OR tourette* OR tic-disorder* OR attention-deficit* OR adhd OR epilep* OR language-disorder* OR neurodevelopment* OR ((neuro OR cognitive OR physical*) W/5 development*)) W/ 6 water)

OR (mortalit* OR death* OR fatalit*) OR (respiratory OR asthma* OR allerg* OR aeroallerg* OR ((breathing OR breathe) W/3 (issue* OR problem* OR difficult* OR struggl* OR trouble))) OR (heat-stress* OR heat-exhaustion OR heat-stroke* OR heatstroke* OR "sun stroke*" OR sunstroke*) OR (cholera* OR diarrhea* OR diarrhoea* OR dysentery OR salmonella OR pneumonia* OR plasmodium* OR babesi* OR hantavirus* OR giardia* OR cryptosporidi* OR leptospiros*) OR (trichinos* OR trichinellosis OR chikungunya OR dengue OR lymphatic-filariasis OR rift-valley-fever OR yellow-fever OR zika OR malaria OR japanese-encephali))))) AND NOT (TITLE-ABS-KEY (mustelid* OR salmon* OR dog OR dogs OR squirrel* OR smoker* OR smoking OR animal* OR tarantula* OR fish* OR spruce* OR moss OR tree OR trees OR cottonwood* OR deer OR moose OR boar OR boars OR aphid* OR bryozoa* OR bird* OR rat OR rats OR mice OR mouse OR rabbit* OR cat OR cats))

Cochrane Library Results = 62

ID Search Hits

#1 (elders OR elderly OR geriatric* OR "old age" OR "senior citizens" OR "older adult*" OR centenarian* OR nonagenarian* OR octogenarian* OR septuagenarian* OR sexagenarian* OR dottering OR decrepit OR tottering OR overaged OR "oldest old" OR pediatric* OR paediatric* OR child* OR newborn* OR congenital* OR infan* OR baby OR babies OR neonat* OR pre-term OR preterm* OR "premature birth*" OR nicu OR preschool* OR pre-school* OR kindergarten* OR kindergarden* OR "elementary school" OR "nursery school*" OR "day care*" OR schoolchild* OR toddler* OR boy O R boys OR girl* OR "middle school*" OR pubescen* OR juvenile* OR teen* OR youth* OR "high school*" OR adolesc* OR pre-pubesc* OR prepubesc* OR immigrant* OR immigration OR emmigrant* OR emmigration OR refugee* OR "asylum seeker*" OR asylee* OR "displaced person*" OR "incomer*" OR "in comer*" OR "new comer*" OR newcomer* OR childbearing OR childbirth OR pregnan* OR fertile OR fecund OR womanish OR womanly OR maternal OR vulnerable):ti,ab,kw (Word variations have been searched) 390575

#2 (Saulteaux or Wakashan or Cree or Dene or Inuit or Inuk or Inuvialuit* or Haida or Ktunaxa or Tsimshian or Gitsxan or Nisga'a or Haisla or Heiltsuk or Oweenkeno or Kwakwaka'wakw or Nuu chah nulth or Tsilhqot'i. or Dakelh or Wet'suwet'en or Sekani or Dunne-za or Dene or Tahltan or Kaska or Tagish or Tutchone or Nuxalk or Salish or Stl'atlimc or Nlaka'pamux or Okanagan or Sec wepmc or Tlingit or Anishinaabe or Blackfoot or Nakoda or Tasttine or Tsuu T'inia or Gwich'in or Han or Tagish or Tutchone or Algonquin or Nipissing or Ojibwa or Potawatomi or Innu or Maliseet or Mi'kmaq or Micmac or Passamaquoddy or Haudenosaunee or Cayuga or Mohawk or Oneida or Onodaga or Seneca or Tuscarora or Wyandot or Aboriginal* or Indigenous* or Metis or red road or "on reserve" or off-reserve or First Nation or First Nations or Amerindian or (urban adj3 (Indian* or Native* or Aboriginal*)) or ethnomedicin. or country food* or residential school* or shaman* or traditional heal* or traditional food* or medicine man or medicine woman or autochtone*):ti,ab,kw 23769

#3 MeSH descriptor: [Indians, North American] explode all trees 241

#4 MeSH descriptor: [Inuits] explode all trees 33

#5 (arborist* or archaeologist* or fruit picker* or farm labor* or farm labour* or berry pick* or cowboy* or cowgirl* or "deck hand" or dog walker* or farmworker* or fisher* worker* or fisherm?n or firefighter* or fire fighter* or farmer* or farmhand* or forester or flagm* or flagwom* or greens keeper* or

grounds keeper* or gardener* or garbagewom* or garbage man or garbage men or garbage women or garbage woman or garbage collector* or garbage worker* or herder* or hunter* or letter carrier* or lifeguard* or landscaper* or mail carrier* or marine biologist* or mariner* or outdoor* work* or outdoor employment* or outdoor construction* or outdoor job* or outside work* or outside employment* or outside construction* or outside job* or "out of doors work*" or "out of doors employment" or "out of doors job*" or playground supervisor* or power line technician* or ski instructor* or ski patrol* or ranger or rangers or "rig hand*" or sailor* or parks worker* or shepherd* or smoke jumper* or stoop labour* or stoop labour * or trapper* or tree planter* or vegetable picker* or wildlife biologist*):ti,ab,kw 2607
#6 #1 or #2 or #3 or #4 or #5 406803
#7 ("climat* change" or global-climate* OR "climate event" or "disaster storm*" or "global warming" or "evironmental change" or "greenhouse gas*" OR "greenhouse effect*" OR "carbon emission*" OR "carbon dioxide emission*" OR "CO2 emission*" or desertifcation OR hurricane* OR cyclone* OR tornado* OR superstorm* OR "dust storm*" OR "storm surge*" OR "ice storm*" OR monsoon* or "extreme heat" or "extreme temperature*" or "extreme cold" or wildfire* or "fire disaster*" or "uncontrolled fire*" or "sea level rise" or "permafrost melt" or "sea level ris*" or "glacial retreat" or "ice cap melt*" or "thermohaline conveyor" or "extreme precipitation" or waterlogging or flood* or "El Nino" or "La Nina" or uv-index or uv-rays or ozone hole):ti 122
#8 (((air or atmosphere or gas*) AND (emission* or immission* or quality or pollut* or vapor* or vapour* or purifi* or fume*))):ti 786
#9 ((afterburning or smog or electrosmog or flaring or ordor or odour or "fly ash" or "fall out" or "acid rain" or "acid precipitation" or ‘ "airborne particulate")):ti,ab,kw 1513
#10 ((dust or ((industrial or traffic or automobile or diesel of vehicle or roadway or motorway or highway) and (emit* or emiss* or vapor* or vapour* or fume*)))):ti 902
#11 (("workplace air" or "factory air" or "airborne pesticide*" or "airborne herbicide*" or "airborne insecticide*" or "insecticide mist*" or "pesticide mist*" or "herbicide mist*")):ti,ab,kw 6
#12 #7 or #8 or #9 or #10 or #11 3308
#13 (((Cambridge or (Halifax or Hamilton or London or Victoria or Waterloo or Welland or Whitby or Windsor)) not (UK or Britain or United Kingdom or England or Australia))):ti,ab,kw11480
#14 canad* or "british columbia" or alberta* or saskatchewan or manitoba* or ontario or quebec or (new brunswick not new jersey) or nouveau brunswick or nova scotia or nouvelle ecosse or prince edward island or newfoundland or labrador or nunavut or nwt or northwest territories or yukon or nunavik or inuvialuit or Abbotsford or Airdrie or Ajax or Aurora or Barrie or Belleville or Blainville or Brampton or Brantford or Brossard or Burlington or Burnaby or Caledon or Calgary or Cape Breton or Chatham Kent or Chilliwack or Clarington or Coquitlam or Drummondville or Edmonton or Fredericton or Fort McMurray or Gatineau or Granby or Grande Prairie or Sudbury or Guelph or Halton Hills or Iqaluit or Inuvik or Kamloops or Kawartha Lakes or Kelowna or Kingston or Kitchener or Langley or Laval or Lethbridge or Levis or Longueuil or Maple Ridge or Markham or Medicine Hat or Milton or Mirabel or Mississauga or Moncton or Montreal or Nanaimo or New Westminster or Newmarket or Niagara Falls or Norfolk County or North Bay or North Vancouver or North Vancouver or Oakville or Oshawa or Ottawa or Peterborough or Pickering or Port Coquitlam or Prince George or Quebec City or Red Deer or Regina or Repentigny or Richmond or Richmond Hill or Saanich or Saguenay or Saint

John or Saint-Hyacinthe or Saint-Jean-sur-Richelieu or Saint-Jerome or Sarnia or Saskatoon or Sault Ste Marie or Sherbrooke or St Albert or St Catharines or St John's or Strathcona County or Surrey or Terrebonne or Thunder Bay or Toronto or Trois-Rivieres or Vancouver or Vaughan or Whitehorse or Winnipeg or Wood Buffalo or Yellowknife:ti,ab 67046

#15 MeSH descriptor: [Canada] explode all trees3409

#16 #13 or #14 or #15 77489

#17 #6 and #12 and #16 62

POSPERO Results =32

#1 ((elders OR elderly OR geriatric* OR "old age" OR "senior citizens" OR "older adult*" OR centenarian* OR nonagenarian* OR octogenarian* OR septuagenarian* OR sexagenarian* OR dottering OR decrepit OR tottering OR overaged OR "oldest old" OR pediatric* OR paediatric* OR child* OR newborn* OR congenital* OR infan* OR baby OR babies OR neonat* OR pre-term OR preterm* OR "premature birth*" OR nicu OR preschool* OR pre-school* OR kindergarten* OR kindergarden* OR "elementary school*" OR "nursery school*" OR "day care*" OR schoolchild* OR toddler* OR boy OR boys OR girl* OR "middle school*" OR pubescen* OR juvenile* OR teen* OR youth* OR "high school*" OR adolesc* OR pre-pubesc* OR prepubesc* OR immigrant* OR immigration OR emmigrant* OR emmigration OR refugee* OR "asylum seeker*" OR asylee* OR "displaced person*" OR "incomer*" OR "in comer*" OR "new comer*" OR newcomer* OR childbearing OR childbirth OR pregnan* OR fertile OR fecund OR womanish OR womanly OR maternal OR vulnerable)) 33229

#2 Saulteaux or Wakashan or Cree or Dene or Inuit or Inuk or Inuvialuit* or Haida or Ktunaxa or Tsimshian or Gitsxan or Nisga'a or Haisla or Heiltsuk or Oweenkeno or Kwakwaka'wakw or Nuu chah nulth or Tsilhqot'i. or Dakelh or Wet'suwet'en or Sekani or Dunne-za or Dene or Tahltan or Kaska or Tagish or Tutchone or Nuxalk or Salish or Stl'atlimc or Nlaka'pamux or Okanagan or Sec wepmc or Tlingit or Anishinaabe or Blackfoot or Nakoda or Tasttine or Tsuu T'inia or Gwich'in or Han or Tagish or Tutchone or Algonquin or Nipissing or Ojibwa or Potawatomi or Innu or Maliseet or Mi'kmaq or Micmac or Passamaquoddy or Haudenosaunee or Cayuga or Mohawk or Oneida or Onodaga or Seneca or Tuscarora or Wyandot or Aboriginal* or Indigenous* or Metis or red road or "on reserve" or off-reserve or First Nation or First Nations or Amerindian 1155

#3 "urban indian*" or "urban native*" or "country food*" or "residential school*" or shaman* or "traditional heal*" or "traditional food*" or "medicine man" or "medicine woman" or autochtone* 109

#4 arborist* or archaeologist* or fruit picker* or farm labor* or farm labour* or berry pick* or cowboy* or cowgirl* or "deck hand" or dog walker* or farmworker* or fisher* worker* or fisherm?n or firefighter* or fire fighter* or farmer* or farmhand* or forester or flagm* or flagwom* or greens keeper* or grounds keeper* or gardener* or garbagewom* or garbage man or garbage men or garbage women or garbage woman or garbage collector* or garbage worker* or herder* or hunter* or letter carrier* or lifeguard* or landscaper* or mail carrier* or marine biologist* or mariner* or outdoor* work* or outdoor employment* or outdoor construction* 611

#5 outdoor job* or outside work* or outside employment* or outside construction* or outside job* or "out of doors work*" or "out of doors employment" or "out of doors job*" or playground 36

#6 power line technician* or ski instructor* or ski patrol* or ranger or rangers or "rig hand*" or sailor* or parks worker* or shepherd* or smoke jumper* or stoop labour* 100

#7 stoop labour * or trapper* or tree planter* or vegetable picker* or wildlife biologist*2

#8 #1 OR #2 OR #3 OR #4 OR #5 OR #6 OR #7 34243

#9 (((Cambridge or (Halifax or Hamilton or London or Victoria or Waterloo or Welland or Whitby or Windsor)) not (UK or Britain or United Kingdom or England or Australia))) 1424

#10 canad* or "british columbia" or alberta* or saskatchewan or manitoba* or ontario or quebec or (new brunswick not new jersey) or nouveau brunswick or nova scotia or nouvelle ecosse or prince edward island or newfoundland or labrador or nunavut or nwt or northwest territories or yukon or nunavik or inuvialuit or Abbotsford or Airdrie or Ajax or Aurora or Barrie or Belleville or Blainville or Brampton or Brantford or Brossard or Burlington or Burnaby or Caledon or Calgary or Cape Breton or Chatham Kent or Chilliwack or Clarington or Coquitlam or Drummondville or Edmonton or Fredericton or Fort McMurray or Gatineau or Granby or Grande Prairie or Sudbury or Guelph or Halton Hills or Iqaluit or Inuvik or Kamloops or Kawartha Lakes or Kelowna or Kingston or Kitchener or Langley or Laval or Lethbridge or Levis or Longueuil or Maple Ridge or Markham or Medicine Hat or Milton or Mirabel or Mississauga or Moncton or Montreal or Nanaimo or New Westminster or Newmarket or Niagara Falls or Norfolk County or North Bay or North Vancouver or North Vancouver or Oakville or Oshawa or Ottawa or Peterborough or Pickering or Port Coquitlam or Prince George or Quebec City or Red Deer or Regina or Repentigny or Richmond or Richmond Hill or Saanich or Saguenay or Saint John or Saint-Hyacinthe or Saint-Jean-sur-Richelieu or Saint-Jerome or Sarnia or Saskatoon or Sault Ste Marie or Sherbrooke or St Albert or St Catharines or St John's or Strathcona County or Surrey or Terrebonne or Thunder Bay or Toronto or Trois-Rivieres or Vancouver or Vaughan or Whitehorse or Winnipeg or Wood Buffalo or Yellowknife 17864

#11 #9 OR #10 18451

#12 (("climat* change" or global-climate* OR "climate event" or "disaster storm*" or "global warming" or "evironmental change" or "greenhouse gas*" OR "greenhouse effect*" OR "carbon emission*" OR "carbon dioxide emission*" OR "CO2 emission*" or desertifcation OR hurricane* OR cyclone* OR tornado* OR superstorm* OR "dust storm*" OR "storm surge*" OR "ice storm*" OR monsoon* or "extreme heat" or "extreme temperature*" or "extreme cold" or wildfire* or "fire disaster*" or "uncontrolled fire*" or "sea level rise" or "permafrost melt" or "sea level ris*" or "glacial retreat" or "ice cap melt*" or "thermohaline conveyor" or "extreme precipitation" or waterlogging or flood* or "El Nino" or "La Nina" or uv-index or uv-rays or ozone hole)):TI 36

#13 ((((air or atmosphere or gas*) AND (emission* or immission* or quality or pollut* or vapor* or vapour* or purifi* or fume*)))):TI 121

#15 (((dust or ((industrial or traffic or automobile or diesel of vehicle or roadway or motorway or highway) and (emit* or emiss* or vapor* or vapour* or fume*))))):TI9

#16 (((afterburning or smog or electrosmog or flaring or ordor or odour or "fly ash" or "fall out" or "acid rain" or "acid precipitation" or "airborne particulate"))):TI 3

#17 (("workplace air" or "factory air" or "airborne pesticide*" or "airborne herbicide*" or "airborne insecticide*" or "insecticide mist*" or "pesticide mist*" or "herbicide mist*"))0

#19 #12 OR #13 OR #14 OR #15 OR #16 OR #17 169

#20 #8 AND #11 and #19 32

PROQUEST Dissertations and Theses Global Results =140

(noft(((elders OR elderly OR geriatric* OR "old age" OR "senior citizens" OR "older adult*" OR centenarian* OR nonagenarian* OR octogenarian* OR septuagenarian* OR sexagenarian* OR dottering OR decrepit OR tottering OR overaged OR "oldest old" OR pediatric* OR paediatric* OR child* OR newborn* OR congenital* OR infan* OR baby OR babies OR neonat* OR pre-term OR preterm* OR "premature birth*" OR nicu OR preschool* OR pre-school* OR kindergarten* OR kindergarden* OR "elementary school*" OR "nursery school*" OR "day care*" OR schoolchild* OR toddler* OR boy OR boys OR girl* OR "middle school*" OR pubescen* OR juvenile* OR teen* OR youth* OR "high school*" OR adolesc* OR pre-pubesc* OR prepubesc* OR immigrant* OR immigration OR emmigrant* OR emmigration OR refugee* OR "asylum seeker*" OR asylee* OR "displaced person*" OR "incomer*" OR "in comer*" OR "new comer*" OR newcomer* OR childbearing OR childbirth OR pregnan* OR fertile OR fecund OR womanish OR womanly OR maternal OR vulnerable))) OR noft(Saulteaux OR Wakashan OR Cree OR Dene OR Inuit OR Inuk OR Inuvialuit* OR Haida OR Ktunaxa OR Tsimshian OR Gitsxan OR Nisga'a OR Haisla OR Heiltsuk OR Oweenkeno OR Kwakwaka'wakw OR Nuu chah nulth OR Tsilhqot'i. OR Dakelh OR Wet'suwet'en OR Sekani OR Dunne-za OR Dene OR Tahltan OR Kaska OR Tagish OR Tutchone OR Nuxalk OR Salish OR Stl'atlimc OR Nlaka'pamux OR Okanagan OR Sec wepmc OR Tlingit OR Anishinaabe OR Blackfoot OR Nakoda OR Tasttine OR Tsuu T'inia OR Gwich'in OR Han OR Tagish OR Tutchone OR Algonquin OR Nipissing OR Ojibwa OR Potawatomi OR Innu OR Maliseet OR Mi'kmaq OR Micmac OR Passamaquoddy OR Haudenosaunee OR Cayuga OR Mohawk OR Oneida OR Onodaga OR Seneca OR Tuscarora OR Wyandot OR Aboriginal* OR Indigenous* OR Metis OR red road OR "on reserve" OR off-reserve OR First Nation OR First Nations OR Amerindian) OR noft("urban indian*" OR "urban native*" OR "country food*" OR "residential school*" OR shaman* OR "traditional heal*" OR "traditional food*" OR "medicine man" OR "medicine woman" OR autochtone*) OR noft(#4 arborist* OR archaeologist* OR fruit picker* OR farm labor* OR farm labour* OR berry pick* OR cowboy* OR cowgirl* OR "deck hand" OR dog walker* OR farmworker* OR fisher* worker* OR fisherm?n orghter* OR fire fighter* OR farmer* OR farmhand* OR forester OR flagm* OR flagwom* OR " greens firefikeeper*" OR "grounds keeper*" OR gardener* OR garbagewom* OR garbage man OR garbage men OR garbage women OR garbage woman OR garbage collector* OR garbage worker* OR herder* OR hunter* OR letter carrier* OR lifeguard* OR landscaper* OR mail carrier* OR marine biologist* OR

mariner* OR outdoor* work* OR "outdoor employment*" OR "outdoor construction*") OR noft("outdoor job*" OR "outside work*" OR "outside employment*" OR "outside construction*" OR "outside job*" OR "out of doors work*" OR "out of doors employment" OR "out of doors job*" OR "playground supervisor*") OR noft("power line technician*" OR "ski instructor*" OR "ski patrol*" OR ranger OR rangers OR "rig hand*" OR sailor* OR "parks worker*" OR shepherd* OR "smoke jumper*" OR "stoop labour*") OR noft("stoop labour*" OR trapper* OR "tree planter*" OR "vegetable picker*" OR "wildlife biologist*")) AND (ab((((Cambridge OR (Halifax OR Hamilton OR London OR Victoria OR Waterloo OR Welland OR Whitby OR Windsor)) NOT (UK OR Britain OR United Kingdom OR England OR Australia)))) OR ti((((Cambridge OR (Halifax OR Hamilton OR London OR Victoria OR Waterloo OR Welland OR Whitby OR Windsor)) NOT (UK OR Britain OR United Kingdom OR England OR Australia)))) OR ti(canad* OR "british columbia" OR alberta* OR saskatchewan OR manitoba* OR ontario OR quebec OR (new brunswick NOT new jersey) OR nouveau brunswick OR nova scotia OR nouvelle ecosse OR prince edward island OR newfoundland OR labrador OR nunavut OR nwt OR northwest territories OR yukon OR nunavik OR inuvialuit OR Abbotsford OR Airdrie OR Ajax OR Aurora OR Barrie OR Belleville OR Blainville OR Brampton OR Brantford OR Brossard OR Burlington OR Burnaby OR Caledon OR Calgary OR Cape Breton OR Chatham Kent OR Chilliwack OR Clarington OR Coquitlam OR Drummondville OR Edmonton OR Fredericton OR Fort McMurray OR Gatineau OR Granby OR Grande Prairie OR Sudbury OR Guelph OR Halton Hills OR Iqaluit OR Inuvik OR Kamloops OR Kawartha Lakes OR Kelowna OR Kingston OR Kitchener OR Langley OR Laval OR Lethbridge OR Levis OR Longueuil OR Maple Ridge OR Markham OR Medicine Hat OR Milton OR Mirabel OR Mississauga OR Moncton OR Montreal OR Nanaimo OR New Westminster OR Newmarket OR Niagara Falls OR Norfolk County OR North Bay OR North Vancouver OR North Vancouver OR Oakville OR Oshawa OR Ottawa OR Peterborough OR Pickering OR Port Coquitlam OR Prince George OR Quebec City OR Red Deer OR Regina OR Repentigny OR Richmond OR Richmond Hill OR Saanich OR Saguenay OR Saint John OR Saint-Hyacinthe OR Saint-Jean-sur-Richelieu OR Saint-Jerome OR Sarnia OR Saskatoon OR Sault Ste Marie OR Sherbrooke OR St Albert OR St Catharines OR St John's OR Strathcona County OR Surrey OR Terrebonne OR Thunder Bay OR Toronto OR Trois-Rivieres OR Vancouver OR Vaughan OR Whitehorse OR Winnipeg OR Wood Buffalo OR Yellowknife) OR ab(canad* OR "british columbia" OR alberta* OR saskatchewan OR manitoba* OR ontario OR quebec OR (new brunswick NOT new jersey) OR nouveau brunswick OR nova scotia OR nouvelle ecosse OR prince edward island OR newfoundland OR labrador OR nunavut OR nwt OR northwest territories OR yukon OR nunavik OR inuvialuit OR Abbotsford OR Airdrie OR Ajax OR Aurora OR Barrie OR Belleville OR Blainville OR Brampton OR Brantford OR Brossard OR Burlington OR Burnaby OR Caledon OR Calgary OR Cape Breton OR Chatham Kent OR Chilliwack OR Clarington OR Coquitlam OR Drummondville OR Edmonton OR Fredericton OR Fort McMurray OR Gatineau OR Granby OR Grande Prairie OR Sudbury OR Guelph OR Halton Hills OR Iqaluit OR Inuvik OR Kamloops OR Kawartha Lakes OR Kelowna OR Kingston OR Kitchener OR Langley OR Laval OR Lethbridge OR Levis OR Longueuil OR Maple Ridge OR Markham OR Medicine Hat OR Milton OR Mirabel OR Mississauga OR Moncton OR Montreal OR Nanaimo OR New Westminster OR Newmarket OR Niagara Falls OR Norfolk County OR North Bay OR North Vancouver OR North Vancouver OR Oakville OR Oshawa OR Ottawa OR Peterborough OR Pickering OR Port Coquitlam OR Prince George OR Quebec City OR Red Deer OR Regina OR Repentigny OR Richmond OR Richmond Hill OR Saanich OR Saguenay OR Saint John OR Saint-Hyacinthe OR Saint-Jean-sur-Richelieu OR Saint-Jerome OR Sarnia OR Saskatoon OR Sault Ste Marie OR Sherbrooke OR St Albert OR St Catharines OR St John's OR Strathcona County OR Surrey OR Terrebonne OR Thunder Bay OR Toronto OR Trois-Rivieres OR Vancouver OR Vaughan OR Whitehorse OR Winnipeg OR Wood Buffalo OR Yellowknife)) AND (ti((("climat* change" OR global-climate* OR "climate event" OR "disaster storm*" OR "global warming" OR "evironmental change" OR "greenhouse gas*" OR "greenhouse effect*" OR "carbon emission*" OR "carbon dioxide emission*" OR "CO2 emission*" OR desertifcation OR hurricane* OR cyclone* OR tornado* OR superstorm* OR "dust storm*" OR "storm surge*" OR "ice storm*" OR monsoon* OR "extreme heat" OR "extreme temperature*" OR "extreme cold" OR wildfire* OR "fire disaster*" OR "uncontrolled fire*" OR "sea level rise" OR "permafrost melt" OR "sea level ris*" OR "glacial retreat" OR "ice cap

melt*" OR "thermohaline conveyor" OR "extreme precipitation" OR waterlogging OR flood* OR "El Nino" OR "La Nina" OR uv-index OR uv-rays OR ozone hole))) OR ti(((((air OR atmosphere OR gas*) AND (emission* OR immission* OR quality OR pollut* OR vapor* OR vapour* OR purifi* OR fume*))))) OR ti((((dust OR ((industrial OR traffic OR automobile OR diesel of vehicle OR roadway OR motorway OR highway) AND (emit* OR emiss* OR vapor* OR vapour* OR fume*)))))) OR ti((((afterburning OR smog OR electrosmog OR flaring OR ordor OR odour OR "fly ash" OR "fall out" OR "acid rain" OR "acid precipitation" OR "airborne particulate")))) OR ti((("workplace air" OR "factory air" OR "airborne pesticide*" OR "airborne herbicide*" OR "airborne insecticide*" OR "insecticide mist*" OR "pesticide mist*" OR "herbicide mist*"))))
