# Supplementary material for: Risk and Resilience: How Is the Health of Older Adults and Immigrant People Living in Canada Impacted by Climate- and Air Pollution-Related Exposures?
Source: Int J Environ Res Public Health. 2021 Oct 9;18(20):10575. doi: 10.3390/ijerph182010575 (PMC8535805; doi:10.3390/ijerph182010575)
Supplement: Supplementary file 1 [file ijerph-18-10575-s001.zip › Tilstra_IJERPH_S2.pdf]

Supplementary Material

The following table is a more detailed summary of the articles included in this scoping review. Significant results pertaining to older adults and immigrants are reported. In some cases there were a large number of measures of association reported, so a written summary is given instead of the values.

Table S2. Climatic and air pollution variables and links to health in older adults and immigrant populations.

Authors	Location, setting, and period	Population (n, sex, age)	Primary exposure variables	Health impact category	Study Design	Relevant contextual factors adjusted for in study	Main findings [95% CI]
				**Meteorological**			
**Vanasse 2017 [29]**	Quebec; urban; 2001 – 2011	112,793 participants; 48.5% male; Age: ≥65 years, mean age 79.8 years (7.9 SD)	Mean temperature	Cardiovascular	Cohort	Sex, comorbidities, season, region	Mean temperature (3 day lag) RR: 0.994 [0.992, 0.996] Atmospheric pressure (7 day lag) RR: 1.045 [1.015, 1.077]
**Bai 2018a [30]**	Ontario; province wide; 1996 – 2013	**Coronary heart disease**: 1,389,057 cases; 65.9 % male; Age: mean age 66.5 (64-82 IQR) **Stroke**: 355,837 stroke cases; 50.8% male; Age: mean age 71.0 (64-82 IQR)	Mean, maximum, and extreme temperatures	Cardiovascular	Cohort	Age, sex, comorbidities	Hot days ≥65 years taking oral anticoagulants RR: 1.48 [1.11, 1.97] ≥65 years not taking oral anticoagulants RR: 0.99 [0.89, 1.10]
**Vida 2012 [31]**	Quebec; urban; 1995 – 2007	347,552 events; Age: <65 years, ≥65 years (distribution NR)	Mean temperature, diurnal temperature change	Mental health	Ecological	Age, region	20^0C IRR: 1.00 [0.98, 1.02] 22.5^0C IRR: 1.03 [1.01, 1.06] 25^0C IRR: 1.09 [1.03, 1.15]

**Wang 2014 [32]**	Ontario; urban; April 2002 – March 2010	271,746 events; Age: 0-14 years, 15-39 years, 50-59 years, ≥60 years (distribution NR)	Minimum, maximum, mean temperature	Mental health	Ecological	Age, sex	High temperature associated with increased risk in ≥60 years.
**Benmarhnia 2017 [33]**	Quebec; urban; 1990 – 2010 (June – August, summers)	Age: <65 years vs. ≥65 years (distribution NR)	Mean temperature (threshold and percentile methods)	Mortality	Ecological	Age	≥65 years RR: 1.16 [1.12, 1.19]
**Henderson 2013 [34]**	British Columbia; province-wide; 1986 – 2010	~4.6 million people; Age: <75 years vs. ≥75 years<br>% ≥75 years 1986/2010:<br>Coast: 4.5/6.6<br>Dry Plateua: 4.7/8.5<br>Mountain 3.0/6.8<br>North 1.3/4.3	Apparent maximum temperature (ºC)	Mortality	Ecological	Age, periods	Coast, ≥75 yrs: 18% [14, 22%] increase
**Vutcovici 2014 [35]**	Quebec; urban; 1984 – 2007	Mean 30.1 (6.9 SD) deaths per day for 8,766 days;<br>Age: ≥65 years	Diurnal temperature change	Mortality	Ecological	None	25th %ile to 75th %ile change:<br>5.12% [0.02, 10.49%] increase<br>75th to 99th %ile change:<br>11.27% [2.08, 1.29%] increase
**Kosatsky 2012 [36]**	British Columbia; urban; 2001 – 2009	398 participants; Age: <65 years, 65-75 years, 75-85 years, ≥85 (distribution NR)	Heatwave	Mortality; cardiovascular; respiratory	Case-only	% below low-income cut-off, population density, age, place of death (home, hospital, residential insitution, other), % ≥65 years	65-74 yrs: 1.47 [1.06, 2.03] *(ref. ≥85)*

**Chen 2016 [37]**	Ontario; province-wide; 1996 – 2010	352,818 cases; 49% male; Age: mean age 76 (68-86 IQR)	Mean temperature	Mortality; cardiovascular; respiratory; diabetes	Case-crossover	Time, age, sex, place of death (long-term care facility, hospital, other), comorbidities	Cold and cardiovascular mortality: ≥65 years: 3.0% [2.0, 5.0%] increase
**Bustinza 2013 [38]**	Quebec; province-wide; 2005 – 2010	~6 million people; Age (2005-2009, 2010): <65 years (178, 240), 65-74 years (132, 160), ≥75 years (450, 639)	Heatwave	Mortality; overall morbidity	Ecological	Age	Crude rates per 100,000 person days ≥75 years Ref: 15.99 (15.34, 16.66) Heatwave: 21.22 (19.64, 22.93)
**Burton 2015 [39]**	Canada wide; 2015 – 2015	NR	Flooding	Mortality; respiratory; mental health	Review	Various	Older adults at risk of adverse health events due to flooding
**Mondor 2015 [40]**	Quebec; urban; 1998 – 2006	136, 323 participants; Age: ≥65 years	Freezing rain, snowstorms	Fall-related injuries	Ecological	Age, sex	Freezing rain All injuries 65-74 IRR: 1.24 [1.06, 1.46] ≥75 IRR: 1.17 [1.02, 1.34] All injuries except hip fractures: 65-74 IRR: 1.28 [1.08, 1.50]
**Modarres 2012 [41]**	Quebec; urban; 1993 – 2004	22,855 events out of 1,077,813 at risk; 24.2% male; Age: 40-74 years, ≥75 years	Minimum, maximum, mean temperature, precipitation, snow depth, daylight hours, air pressure	Fall-related injuries	Ecological	Age, sex, seasonality, factors	Decreasing temperature, rainfall, and hours of sunshine and increased snow, was associated with hip fracture in older adults.

**Modarres 2014 [42]**	Quebec; urban; 1993 – 2004	22,850 hip fractures; 24.2% male; Age: 40-74 years, ≥75 years	Minimum, maximum, mean temperature, precipitation, snow depth, daylight hours, air pressure	Fall-related injuries	Ecological	Sex, age, number of days	Maximum pressure and daylight hours were important predictors for older adults. Rainfall depth was an important predictor for older males.
**Auger 2017 [43]**	Quebec; province-wide; April 2006 – September 2013	14,302 participants; 58.8% male; Age: <55 years (28%), 55-64 years (27%), 65-74 years (26%), ≥75 years (19%)	Mean temperature	Ocular	Case-crossover	Age, sex	65-74 years: OR 1.98 [1.30, 3.02]
**Laverdière 2016 [44]**	Quebec; urban; 2006 – 2010	1,233 participants; 48% males; Age: 68-72 years, 73-77 years, 78-82 years (distribution NR)	Daily maximum temperature ≥30^{0}C	Overall morbidity	Cohort	Household income < $20,000, Material and Social Deprivation Index, social isolation, social participation, air conditioning, autonomy, mental and physical disorders, medication, urban heat island	Emergency department presentations AOR: 2.6 [2.0, 3.5] Hospitalizations AOR: 1.7 [1.1, 2.6]
**Bélanger 2014* [45]**	Quebec; urban; 2010 – 2011	3485 participants; 54.2% male; Age: 18-35 years (17.6%) , 35-44 years (13.4%), 45-54 years (18.5%), 55-64 years (21.3%), ≥65 years (29.3%)	Hot and humid days	Overall morbidity	Cross-sectional	Household type, length of residence in Canada, language, education, household income, employment, relative economic status, air conditioning, age, sex, SES factors, comorbidities	Low income ≥65 years (multivariate) OR: 1.5 [1.1–2.1] Foreign born (bivariate) OR: 0.8 [0.6, 0.9]

**Stapleton 2014 [46]**	Ontario; not stated; published 2014	24 participants Age: 12 younger participants, mean age 21 years (3 SD); 12 older participants, mean age 65 years (5 SD)	Hot-dry and hot-humid conditions	Overall morbidity	Non-randomized experimental study	None	In both hot-dry and hot-humid conditions, older adults showed a significant increase in body heat content compared to younger adults.
**Tajmir 2013 [47]**	Ontario; not-stated; published 2013	18 participants (10 younger; 20% male; mean age 26 (2.4 SD); 8 older; 62.5% male; mean age 68 (4.4 SD)	Temperature categories; 23^0C vs. 1^0C	Overall morbidity	Non-randomized experimental study	NA	Older adults had greater impairment of manual movements in cold conditions and reduced sensitivity to decreasing temperatures.
**McTavish 2018 [48]**	Ontario; province-wide; 2005 – 2012	52,913 cases and 174,222 controls; 51.5% male; median age 80 (74-85 IQR), >79 years (51.3%)	Maximum temperature	Renal	Case-control	Neighborhood level income, older age subsets, heat and humidex periods	OR: 1.11 [1.00-1.23]
**Ordon 2015 [49]**	Ontario; province-wide; 2002 – 2013	423,396 participants; 73% male; 18-39 years (31%), 40-49 years (25%), 50-59 years (22%), 60-69 years (13%), >70 years (8%)	Minimum, maximum, mean temperature, extreme temperatures	Renal	Ecological	Income, region of residence, age, sex, comorbid conditions	Heat (90th vs 10th %ile) 60-69 yrs RR: 1.31 [1.02, 1.68] Extreme heat (99th vs 10th %ile) 60-69 yrs RR: 1.44 [1.06, 1.96]
			**Meteorological and Air pollution**				
**Krstic 2011 [50]**	British Columbia; urban; 2004 – 2006	≥65 years	Steadman's apparent temperature, $PM_{2.5}$	Cardiovascular; respiratory	Cross-sectional	Number of days	High and low temperatures associated with increased mortality.
			**Air pollution**				

**Parent 2013* [51]**	Quebec; urban; 2005 – 2008	803 cases and 969 controls; male only; Age: <60 years (21.3%), 60<66 years (28.1%), 66<71 years (24.5%), >71 years (26%)	NO_2	Cancer	Case-control	Annual household income, education, % recent immigrants, smoking and lifestyle habits, severity and screening of cancer, residence	3rd quartile recent immigrants OR: 1.42 [1.09, 1.86]
**Goldberg 2018 [52]**	Quebec; urban; 1996 – 1997	375 cases and 413 controls; female only; Age range: 50-70 years	UFP	Cancer	Case-control	Education, ethnicity, official language, neighborhood income, unemployment rate, education, length of residence <10 years vs. ≥10 years, hormonal receptor status	Positive but insignificant associations
**Goldberg 2017 [53]**	Quebec; urban; 2008 - 2011	681 cases and 596 controls; female only; Age: mean age cases 62.23 years (SD NR), controls 61.03 years (SD NR)	NO_2, UFP	Cancer	Case-Control	Education, ethnicity, official language, neighborhood income, unemployment rate, education, % recent immigrants, length of residence <10 years vs. ≥10 years, hormonal receptor status	Positive but insignificant associations
**Chen 2014 [54]**	Alberta; urban; April 1998 – March 2002	5229 participants; 51% male; Age: 25-44 years (4.1%), 45-64 years (23.7%), 65-74 years (27.1%), 75-84 years (31.1%), ≥85 years (13.5%)	AQHI, NO_2, O_3, $PM_{2.5}$	Cardiovascular	Case-crossover	Age, sex	≥75 years (1-72 hour lag) AQHI OR: 1.30 [1.10, 1.54] Single pollutant model CO OR: 1.50 [1.19, 1.90]

							Multi pollutant model NO_2 OR: 1.70 [1.24, 2.34]
**Wang 2015 [55]**	Alberta; province-wide; 1999 – 2009	25,894 events; 67% male; Age: <65 years, ≥65 years	CO, NO_2, NO, O_3, $PM_{2.5}$	Cardiovascular	Case-crossover	Age, sex, comorbidities	Observed positive associations with NO_2 and NO and negative associations with CO in ≥65 years.
**Weichenthal 2017 [56]**	British Columbia; urban; 2008 – 2015	2,881 events; 68% male; <65 years (45.9%), ≥65 years (54.1%)	$PM_{2.5}$, NO_2, O_3	Cardiovascular	Case-crossover	Age, sex, city	≥65 years $PM_{2.5}$ (3-day mean) OR: 1.06 [1.03, 1.08]
**Gan 2011 [57]**	British Columbia; urban; 1994 – 2002	452,735 participants; 45.9% male; Age: mean age 58.9 (10.5 SD), <60 years, 60-69 years, ≥70 years	BC, NO, NO_2, $PM_{2.5}$	Cardiovascular	Cohort	Neighborhood income, age, sex, comorbidity	BC associated with increased risk of mortality in ≥60 years and hospitalizations in 60-69 years.
**Shin 2019 [58]**	Ontario; province wide; 2001 – 2015	5,071,956 participants; Age: 35-44 years (32%), 45-54 years (28%), 55-64 years (18%), 65-74 years (14%), 75-85 years (8%)	NO_2, O_3, O_x, $PM_{2.5}$	Cardiovascular	Cohort	Household income, age, sex, comorbidities, stroke type	All pollutants were associated with increased risk of cardiovascular outcomes.

**Bai 2019 [59]**	Ontario; province-wide; April 2001 – December 2015	Congestive heart failure: 50,062,146 participants; 48% male; Age: mean age 53.3 (13.0 SD); 35-44 years (5%), 45-54 years (12%), 55-64 years (20%), 65-74 years (33%), 75-85 years (30%); Acute myocardial infarction: 5,141,172 participants; 47.7% male; Age: mean age 53.6 (13.2 SD); 35-44 years (12%), 45-54 years (21%), 55-64 years (21%), 65-74 years (25%), 75-85 years (21%)	$PM_{2.5}$, NO2, O_3, O_x	Cardiovascular	Cohort	Neighborhood level education, unemployment, % recent immigrants, regional differences, Ontario Marginalization Index, access to health care, % visible minorities, age, sex, comorbidities, income level, Ox	All pollutants associated with increased risk in ≥65 years.
**Shin 2018a [60]**	Alberta, British Columbia, Manitoba, Newfoundland and Labrador, Nova Scotia, Ontario, Quebec, Saskatchewan; urban; 1996 – 2012	52-54% of Canadian population during the study period; Age: <50 years, ≥50 years, ≥65 years	O_3	Cardiovascular	Ecological	Age, sex, calendar time	No significant associations ≥50 years.

**Shin 2020 [61]**	Alberta, British Columbia, Manitoba, Newfoundland and Labrador, Nova Scotia, Ontario, Quebec, Saskatchewan; urban; 1984 – 2012	~53% of the Canadian population during the study period; 51% male; Age: 1-65 years (91-86%), >65 years (9-14%)	O_3	Cardiovascular	Ecological	Age, sex, season	≥65 years: 0.8% [0.2, 1.3%] increase
**Stieb 2017 [62]**	Ontario; rural; 2013 – 2014	2013: 36 participants; 44.4% male; Age: 55-59 years (30.6%, 60-64 years (44.4%), 65-69 years (16.7%), ≥70 (8.3%); 2014: 41 participants; 34.1% male; 55-59 years (19.5%), 60-64 years (29.3%), 65-69 years (31.7%), ≥70 years (22%)	AQHI, CO, NO_2, O_3, $PM_{2.5}$, SO_2	Cardiovascular	Panel study	Sex, statin use	AQHI, $PM_{2.5}$, and O_3 associated with subclinical adverse cardio-respiratory effects.
**Stieb 2018 [63]**	British Columbia; urban; 2014 – 2015	2014: 36 participants; 44.4% male; Age: 55-59 years (16.7%), 60-64 years (41.7%), 65-69 years (25.0%), ≥70 years (16.7%); 2015: 34 participants; 32.7% male; Age: 55-59 years (23.5%), 60-64 years (44.1%), 65-69 years (17.6%), ≥70 years (14.6%)	AQHI, CO, NO_2, O_3, $PM_{2.5}$, SO_2	Cardiovascular	Panel study	Sex, statin use	AQHI, $PM_{2.5}$, O_3 and O_x associated with subclinical cardio-respiratory effects.

**Stieb 2019 [64]**	Ontario; urban; 2015	Exercise indoors during poor air quality group: 37 participants; 45.9% male; Age: mean age 66 years (55-81 range); Control group: 35 participants; 42.9% male; Age: mean age 65 years (55-79 range)	AQHI, CO, NO_2, O_3, $PM_{2.5}$	Cardiovascular	Randomized control trial	None	AQHI and $PM_{2.5}$ associated with subclinical cardio-respiratory effects and markers of oxidative stress.
**Bai 2018b [65]**	Ontario; urban; 1996 – 2012	Hypertension: 893,499 participants; 48% male; Age: mean age 48.6 (SD 14.3); Diabetes: 1,056,012 participants; 47% male; Age: mean age 51.1 (15.3 SD)	UFP, NO_2	Cardiovascular; diabetes	Cohort	Neighborhood level education, unemployment rate, % recent immigrants, average household income, age, sex, comorbidities	UFP and NO_2 associated with diabetes and hypertension; NO_2 negatively associated with hypertension in 60-74 years.
**Henderson 2011 [66]**	British Columbia; southeastern corner; July – September 2003	281,711 participants; 46% male; Age: median age 45 (23-62 IQR); 0<5 years (4.3%), 5<10 years (5.0%), 10<20 years (12.3%), 20<30 years (9.5%), 30<40 years (11.5%), 40<50 years (15.4%), 50<60 years (14.3%), 60<70 years (11.8%), 70<80 years (10.3%), ≥80 years (6.3%)	PM_{10}	Cardiovascular; respiratory	Cohort	Neighborhood income, age, sex, number of physician visits in the previous year (preexisting smoke sensitivity)	PM_{10} associated with increased respiratory risk in 60-70 years and ≥80 years.

**Crouse 2015 [67]**	Alberta, British Columbia, Manitoba, Ontario, Quebec; urban; 1984 – 2006	735,590 participants; 48% male; 25-34 years (25.8%), 35-44 years (24.6%), 45-54 years (17.3%), 55-64 years (14.5%), 65-74 years (11.5%), ≥75 years (6.3%)	NO_2	Cardiovascular; respiratory; diabetes; mortality	Cohort	% immigrant status, visible minority status, marital status, employment, education, household income, age, local climate	Non-accidental mortality 60-79 yr HR: 1.06 [1.04, 1.08]
**Farhat 2013 [68]**	Alberta, British Columbia, Manitoba, Newfoundland and Labrador, Nova Scotia, Ontario, Quebec; urban; 1980 – 2001	1,564,583 deaths; Age: <75 years (52%), ≥75 years (48%)	O_3, $PM_{2.5}$	Cardiovascular; respiratory; mortality	Ecological	29 city-level variables on demographic, SES, health care, and lifestyle factors including male unemployment, % manufacturing, % of population stressed, and area of the city	O_3 and $PM_{2.5}$ were associated with increased risk in those ≥75 years.
**Goldberg 2013 [69]**	Quebec; urban; 1990 – 2003	158,350 participants; ≥65 years	CO, NO_2, O_3, $PM_{2.5}$, SO_2	Mortality; cardiovascular; cancer; respiratory; diabetes	Cohort	Seasonality	Positive associations between all pollutants and non-accidental, cardiovascular and diabetes related mortality
**Vanos 2013 [70]**	Alberta, British Columbia, Manitoba, Newfoundland and Labrador, Ontario, Quebec; urban; 1981 - 1999	Age: <65 years, 65-74 years, 75-84 years, ≥85 years (distribution NR)	CO, NO_2, O_3, SO_2	Mortality	Cross-sectional	Age	All pollutants associated with increased risk of mortality in ≥85 years.

**de Roos 2014 [71]**	British Columbia; urban; 1994 - 2002	678,361 participants; 47.6% male; Age: 45-54 years (40.2%), 55-64 years (25%), 65-74 years (21.4%), 75-84 years (13.4%)	Proximity to roads, BC, CO, NO, NO_2, O_3, $PM_{2.5}$, PM_{10}	Musculo-skeletal	Nested case-control	Neighborhood income level, age, sex	O_3 associated with increased risk in ≥65 years.
**Chen 2017a [72]**	Ontario, province-wide; April 1996 – March 2013	2,066,639 participants; 46.8% male; Age: mean age 66.8 (8.2 SD); 55-64 years (44.6%), 65-74 years (34.1%), 75-85 years (20.7%)	NO_2, O_3, $PM_{2.5}$	Neurological	Cohort	Household income, education, Ontario Marginalization Index, regional differences, density of healthcare specialists, region, sex, comorbidities	$PM_{2.5}$ and NO_2 associated with increased risk of dementia in those 55-85 years.
**Chen 2017b [73]**	Ontario; province-wide; 2001 – 2013	2,194,519 participants; 46.6% male; Age: 55-85 years	Proximity to roads, $PM_{2.5}$, NO_2	Neurological	Cohort	Unemployment, % recent immigrants, education, neighborhood income, sex, comorbidities	Closer proximity to roads, $PM_{2.5}$ and NO_2 associated with increased risk of dementia, $PM_{2.5}$ associated with increased risk of Parkinson's.
**Shin 2018b [74]**	Ontario; province-wide; 1996 – 2012	Parkinson's disease and dementia: 2,165,269 participants; 46.8% male; Age: mean age 66.8 (8.2 SD)	NO_2, O_3, $PM_{2.5}$	Neurological	Cohort	Household income, education, Ontario Marginalization Index, regional differences, density of healthcare specialists, region, comorbidities	$PM_{2.5}$ HR: 1.04 [1.01, 1.07]<br>O_3 HR: 1.04 [1.01, 1.07]
**Neupane 2010 [75]**	Ontario; urban; 2003 – 2005	365 cases and 494 controls; Age: ≥65 years	NO_2, $PM_{2.5}$, SO_2	Respiratory	Case-control	Education, occupational exposure, sex, comorbidities, modified Barthel Index	NO_2 (IDW) OR: 2.30 [1.25, 4.21]<br>NO_2 (SPL) OR: 2.19 [1.25, 3.83]<br>$PM_{2.5}$ (IDW) OR: 2.26 [1.20, 4.24]

**Szyszkowicz 2014 [76]**	Ontario; urban; 2003 – 2006	6,697 events; 45.1% male; Age: 2-14 years, (32.1%), 15-39 years, (39.3%), 40-59 years (19.6%), ≥60 years (9%)	AQHI, NO_2, O_3, $PM_{2.5}$	Respiratory	Case-crossover	Age, season	No significant associations in ≥60 years observed.
**Lavigne 2012 [77]**	Ontario; urban; April 2002 – March 2009	3,728 participants; 45.3% male; Age: 2-14 years (33.1%), 15-39 years (40.3%), 40-59 years (19.2%), ≥60 years (7.4%)	NO_2, CO, $PM_{2.5}$, SO_2	Respiratory	Case-crossover	Age, seasons	Cold season O_3 ≥60 yrs OR: 1.60 [1.04, 2.45]
**Gan 2013 [78]**	British Columbia; urban; 1994 – 2002	467,994 participants; 47% male; Age: mean age 60 (11 SD) years	BC, NO, NO_2, $PM_{2.5}$	Respiratory	Cohort	Neighborhood income, age, sex, comorbidity	COPD hospitalization ≥65 yrs RR: 1.06 [1.02, 1.10]
**To 2013 [79]**	Ontario; province-wide; 2003 – 2006	~1.5 million persons with asthma during the study period; Age (outpatient, ED visits, and hospital admissions rate per 1000 persons): 0-4 years (1759, 175, 42), 5-9 years (623, 44, 7), 10-19 years (315, 23, 2) , 20-59 years (527, 35, 3), ≥60 years (694, 27, 4)	AQHI, NO_2, O_3, $PM_{2.5}$	Respiratory	Ecological	Region of residence, age, season	AQHI was associated with asthma in ≥60 years.
**Ward 2015 [80]**	Ontario; province-wide; 2000 – 2006	0-19 years, 20-64 years, ≥65 years (distribution NR)	AQI, CO, O_3, $PM_{2.5}$	Respiratory	Ecological	Age, comorbidities	No significant associations observed in ≥65 years.

* Reported results specific to immigrant populations. AQHI – Air Quality Health Index; AQI – Air Quality Index; BC – Black Carbon; UFP – Ultrafine particles (≤0.1 μm diameter)
